# Best Practices for Variable-Temperature Electrochemistry Experiments and Data Reporting

**DOI:** 10.1021/acsenergylett.5c00308

**Published:** 2025-03-07

**Authors:** Anyesh De, Mamta Dagar, Bryce Kneer, James Kim, Agnes E. Thorarinsdottir

**Affiliations:** Department of Chemistry, University of Rochester, Rochester, New York 14627, United States

Electrochemistry has emerged as a cornerstone in creating sustainable energy solutions owing to the ability of electrochemical technologies and devices to harness renewable energy sources, such as solar and wind, and converting them into usable forms of energy. Multiple parameters, such as electrolyte properties, electrode material and morphology, temperature, and applied potential (or current), influence the outcome of electrochemical reactions and the efficiency of electrochemical processes. While temperature has historically been a relatively underexplored parameter, there has been a recent upsurge in reports on variable-temperature electrochemistry. Variable-temperature electrochemical measurements are especially attractive for the design of thermoelectrochemical devices^1−5^ and electrochemical sensors,^6,7^ for optimizing electrochemical reaction conditions,^8^ as well as for providing valuable information about the thermodynamics of electron-transfer reactions, including thermodynamic parameters and equilibrium constants for a range of systems (Figure 1).^9−16^

**Figure 1:**
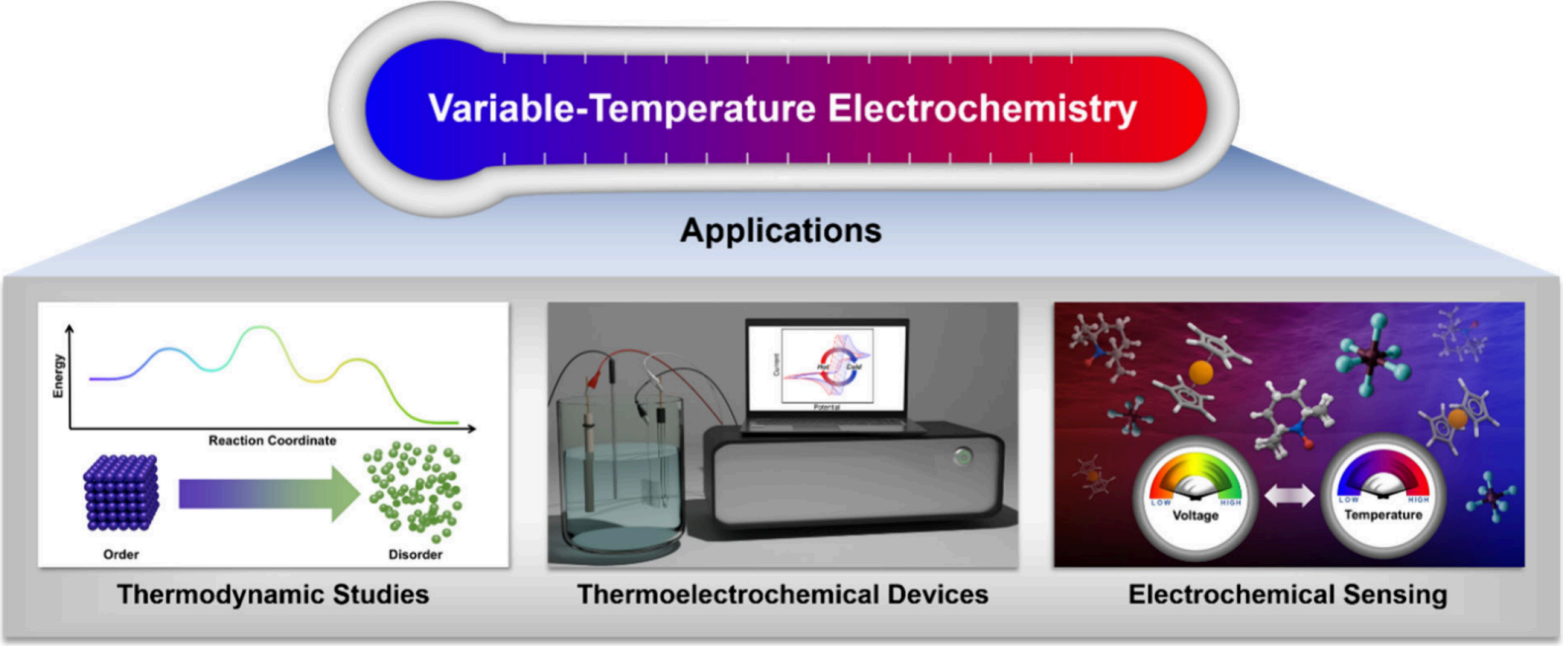
Overview of select applications of variable-temperature electrochemistry.

Given the utility of variable-temperature electrochemical measurements in garnering quantitative thermodynamic information about redox processes, it becomes imperative to understand the correlation between temperature and redox entropies. The temperature dependence of the electrochemical potential (*E*) for a generic cathodic half reaction for solution-phase species at steady state given in eq 1 is linearly correlated with the change in entropy for the half reaction (Δ*S*_redox_) as shown in eq 2, where *n* is the number of electrons transferred, *T* is the temperature, and *F* is Faraday’s constant.^1,17,18^
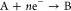
1
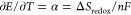
2The temperature dependence of the electrochemical potential is also defined as the temperature coefficient,^19^ and denoted with the parameter α (eq 2). The sign of the temperature coefficient is the same as the sign of the entropy change of the given electrochemical reaction. A positive temperature coefficient indicates an increase in electrochemical potential with increasing temperature, whereas a negative temperature coefficient signifies a decrease in electrochemical potential with increasing temperature.

As the assessment of temperature coefficients for electrochemical half reactions provides the underpinning of variable-temperature electrochemistry, it is critical that such measurements are reliable and reproducible across researchers working in the field of electrochemistry. Unfortunately, reports on temperature coefficient measurements for half-cell reactions frequently lack experimental details that make reproducing and interpreting the published results challenging. To address this issue, the goal of this Viewpoint is to provide general guidelines for conducting variable-temperature electrochemistry experiments and reporting the corresponding data. It is our hope that these guidelines will serve as a benchmark procedure for variable-temperature electrochemistry widely adopted in the electrochemistry community.

In this Viewpoint, we first emphasize proper nomenclature in the field of variable-temperature electrochemistry and discuss commonly analyzed redox systems. Subsequently, we discuss the execution of variable-temperature electrochemical measurements and factors that impact such measurements. This discussion will be illustrated using results from the literature and experiments conducted in our laboratory. Finally, we will provide recommendations for best practices of measuring and reporting variable-temperature electrochemical data.

## Proper Nomenclature

The thermoelectric effect—the direct conversion of a temperature difference to electrochemical potential—has traditionally been observed and utilized in inorganic semimetals and semiconductors.^20,21^ However, ionic thermoelectric materials, which rely on the thermodiffusion and/or the thermogalvanic effect, have gained increased interest for converting a temperature gradient to electrochemical energy owing to the large intrinsic potential difference produced on the order of 1–10s of mV K^–1^ (as compared to 10–100s μV K^–1^ for solid-state thermoelectrics) and operation at temperatures below 200 °C.^5,22,23^ For solid-state systems, the proportionality between the change in potential and change in temperature is given by the Seebeck coefficient (*S*_e_) (eq 3),
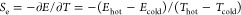
3which is negative for negatively charged carriers and positive for positively charged carriers.^24^ The thermodiffusion effect—also known as the Soret effect or the ionic Seebeck effect—describes the entropic transport of ions from a hot region to a cold region, leading to unequal ion concentrations at the hot and cold electrodes that result in an electric field. This phenomenon is analogous to the Seebeck effect in solid-state materials and can be described by eq 3 upon replacing the electronic Seebeck coefficient (*S*_e_) with the ionic Seebeck coefficient (*S*_td_ or *S*_i_). In contrast, the thermogalvanic effect harvests the reaction entropy of ionic redox couples by maintaining the two electrodes at different temperatures.^5,22,23^ For the cathodic half reaction displayed in eq 1, the temperature dependence of the generated electrochemical potential is given by eq 2, where Δ*S*_redox_ = *S*_B_ – *S*_A_, ∂*E* = *E*_hot_ – *E*_cold_, and ∂*T* = *T*_hot_ – *T*_cold_. For a redox couple with a negative temperature coefficient (*S*_A_ > *S*_B_), oxidation occurs at the hot electrode and reduction at the cold electrode. For a redox couple with a positive temperature coefficient (*S*_B_ > *S*_A_), this process is reversed with oxidation and reduction occurring at the cold and hot electrodes, respectively. Importantly, as is evident by comparing eqs 2 and 3, the temperature coefficient (α) and the Seebeck coefficient (*S*_e_ or *S*_td_/*S*_i_) have the opposite sign convention. This indicates that a redox couple with a positive temperature coefficient would have a negative Seebeck coefficient and vice versa. However, we strongly recommend against using the term “Seebeck coefficient” to describe the temperature dependence of the electrochemical potential for thermogalvanic systems owing to the different mechanism giving rise to the observed phenomenon as compared to solid-state and ionic thermoelectrics. As such, we encourage researchers to refer to temperature coefficients (α), electronic Seebeck coefficients (*S*_e_), and ionic Seebeck coefficients (*S*_td_ or *S*_i_) for thermogalvanic, solid-state thermoelectric, and ionic thermoelectric (or thermodiffusion) systems, respectively. In addition, the word “thermopower” can be used as a general term to describe the temperature dependence of the electrochemical potential induced by any of these effects, with sign conventions clearly stated.^5,22,23^

## Properties of Redox Systems

Multiple redox systems that exhibit the thermogalvanic and/or the thermodiffusion effect have been investigated using variable-temperature electrochemistry. While thermodiffusion systems have provided some of the highest thermopower values reported to date,^22,25−27^ such systems cannot be operated continuously, making them unsuitable for certain applications (e.g., desalination), and we focus our discussion herein on solution-phase redox reactions that rely on the thermogalvanic effect and operate reversibly at steady-state conditions. For homogeneous liquid-phase systems, interactions between the redox-active analyte and surrounding solvent molecules and electrolyte ions are typically the largest contributors to the entropic change associated with the electron-transfer reaction.^1^ So long as low-viscosity solvents such as water and acetonitrile are used, the thermodiffusive contribution from the redox-active analyte and supporting electrolyte ions to the overall thermopower is on the order of ∼10–100s μV K^–1^, much smaller than the temperature coefficient of redox couples (a few mV K^–1^), owing to relatively fast convection.^5,22,23^ For example, the ionic Seebeck coefficient for NaCl in water (at infinite dilution limit) is estimated to be 51 μV K^–1^,^28,29^ while the temperature coefficient of the [Fe(CN)_6_]^3–^/[Fe(CN)_6_]^4–^ couple in water is −1.4 mV K^–1^.^1^ As such, the temperature coefficient of most thermogalvanic systems may be described by eq 2. We note that different additives, including salts and gelators, can be introduced to increase the solution viscosity and generate gel-based systems with high thermopower thanks to synergistic thermogalvanic and thermodiffusion effects.^22,30^ Furthermore, different strategies including harnessing entropy-driven liquid–gas^31^ and other volume phase transition,^32^ thermosensitive crystallization,^33,34^ thermosensitive host–guest chemistry,^35,36^ and proton solvation entropy^37,38^ have been employed to boost the temperature coefficient of thermogalvanic systems. As many redox systems that exhibit the thermogalvanic effect are multicomponent, a detailed understanding of a given electrochemical system is critical prior to quantifying the temperature dependence of its electrochemical potential to identify suitable methods and conditions for performing such analysis and understanding potential limitations. We encourage researchers to evaluate the following using standard analytical and electroanalytical techniques.

- *Thermal and Solution Stability.* Identify the temperature range and solvent(s) in which the redox-active analyte of interest is chemically and electrochemically stable. Such evaluations may for example be performed using NMR spectroscopy or UV–visible absorption spectroscopy in conjunction with cyclic voltammetry at varying temperatures in different solvents.
- *Electrochemical Stability.* Assess the stability of different charge states of the redox-active analyte of interest through controlled potential electrolysis experiments over extended periods of time. Such experiments may for example help in identifying phase-change reactions involving precipitation.
- *Kinetic Stability.* Identify the kinetics of chemical and electrochemical reactions associated with the redox system of interest. Assess if the redox-active analyte is resistant to chemical and electrochemical changes over the time course of the electrochemical experiment. Time-dependent electrochemical measurements, including variable-scan-rate cyclic voltammetry and controlled potential/current vs time experiments may inform kinetic stability in the relevant time scale. For instance, the intensity ratios of the anodic and cathodic peak currents in the voltammograms collected at variable scan rates can inform the rate of electron-transfer reactions and help determine any operable kinetic constraints.
- *Chemical Equilibria.* Identify the chemical equilibria that apply to the redox system of interest. Assess whether homogeneous liquid-phase reactions or multiphase processes are involved, and determine if these reactions are sensitive to the solution proton activity. Standard analytical techniques used to study liquids, gases, and solids may be employed for such assessments. Electrochemical measurements in solutions of variable proton activity can shed light on the sensitivity of the given electron-transfer reactions toward protons, as is the case for proton-coupled electron-transfer reactions. Influence of other ions, such as those of the supporting electrolyte, may be investigated in a similar manner.

## Current Experimental Setups and Measurements

**Figure 2:**
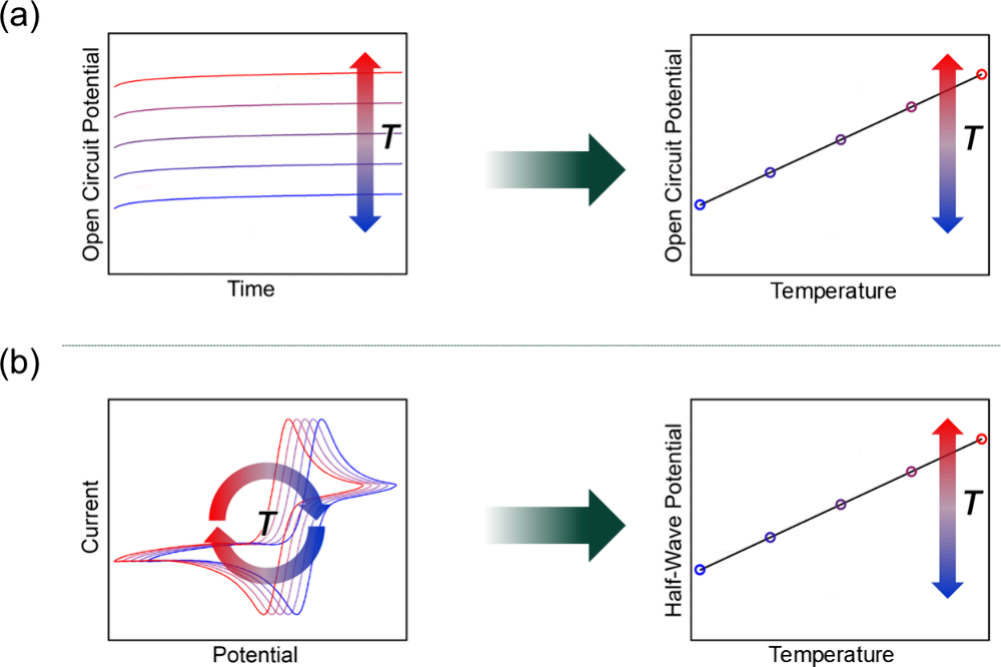
Schematic representations of variable-temperature open circuit potential (a) and cyclic voltammetry (b) data (left) and corresponding plots of open circuit potential or half-wave potential vs temperature obtained under isothermal conditions (right). Note that for analogous nonisothermal measurements, the *x*-axis in the plots on the right would be temperature difference instead of temperature.

Two electrochemical techniques are primarily used for assessing the temperature dependence of the electrochemical potential for a given redox couple: variable-temperature cyclic voltammetry (VT-CV)^10,16,17,39^ and variable-temperature open circuit potential (VT-OCP)^16,40^ measurements (Figure 2). In the VT-CV method, a cyclic voltammogram is collected of the reduced or oxidized form of the redox-active analyte of interest at varying temperatures and the temperature dependence of the formal potential (*E*°′) is analyzed. Note that the formal potential may be estimated as the half-wave potential (*E*_1/2_) so long as the diffusion coefficients of the two redox forms are similar.^41^ In contrast, the VT-OCP technique requires equimolar solutions of the oxidized and reduced forms of a redox couple and entails collecting OCP values (*E*_OCP_) as a function of time (for a few minutes) at a series of temperatures, and subsequent analysis of the temperature dependence of the *E*_OCP_ values.^16,40^

**Figure 3:**
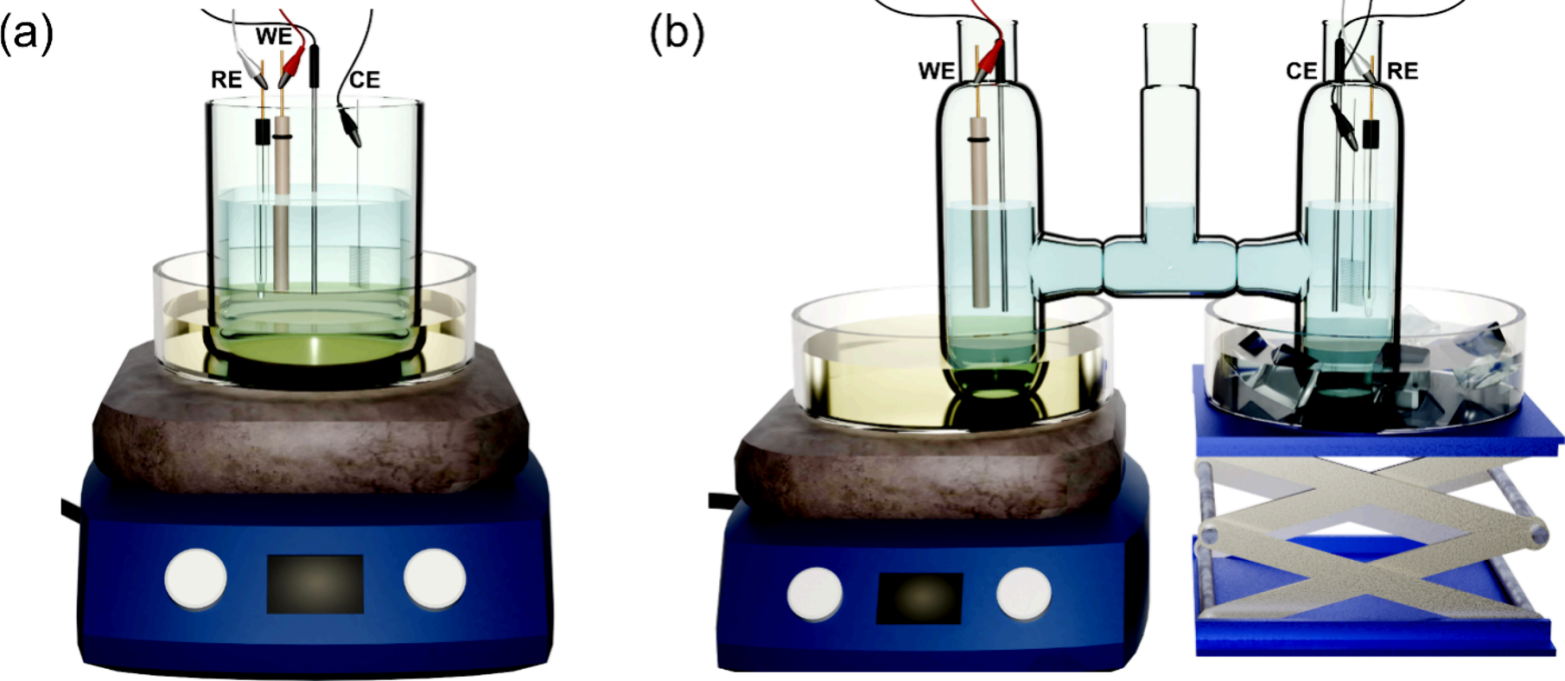
Schematic representations of experimental setups used for variable-temperature electrochemical studies: isothermal (a) and nonisothermal (b) setups. WE, CE, and RE denote working electrode, counter electrode, and reference electrode, respectively. The additional black-capped metal rods immersed in the solutions denote temperature probes.

**Figure 4:**
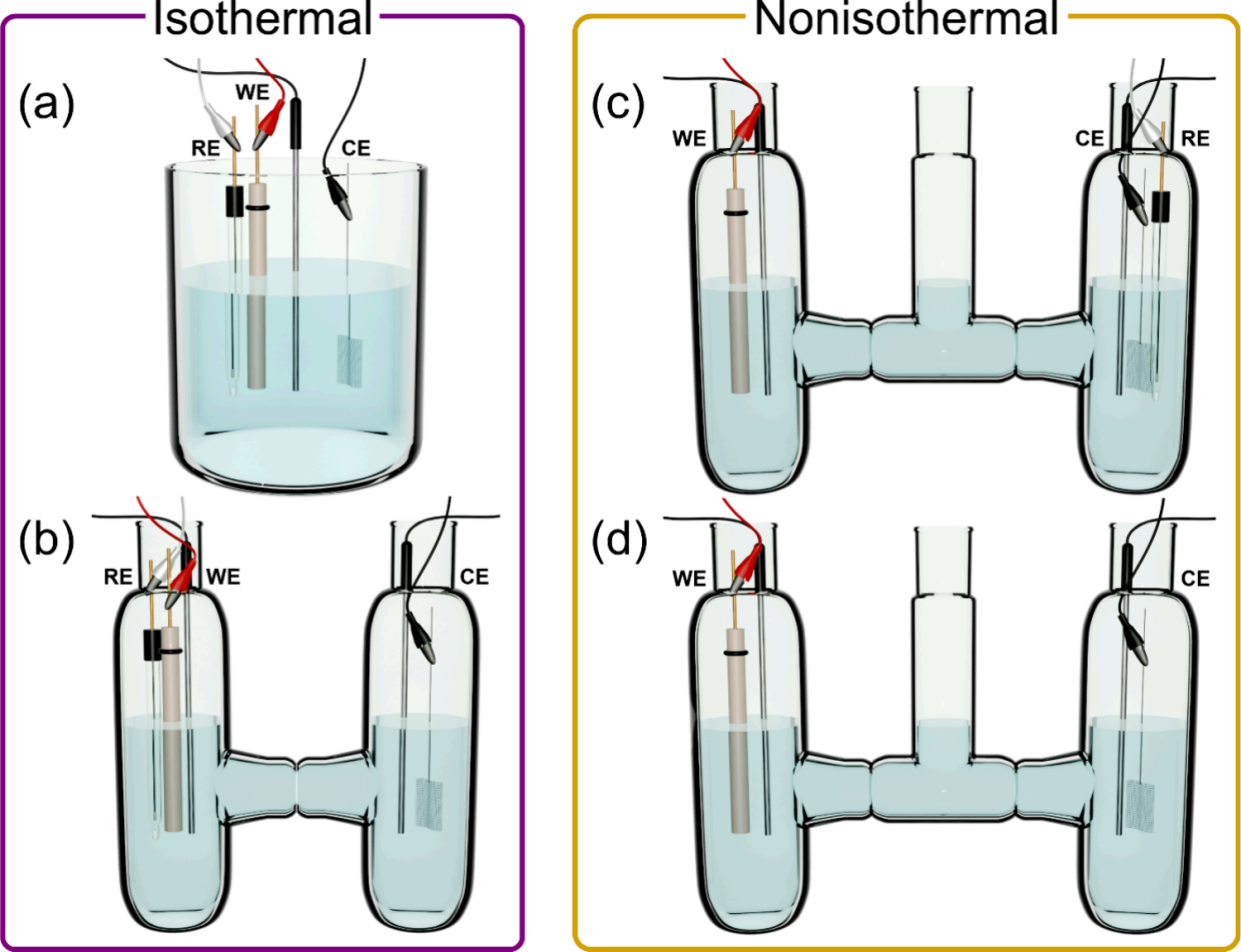
Schematic representations of electrochemical cell configurations used for different types of isothermal (a, b) and nonisothermal (c, d) variable-temperature electrochemical measurements. WE, CE, and RE denote working electrode, counter electrode, and reference electrode, respectively. The additional black-capped metal rods immersed in the solutions denote temperature probes. Connections between glass compartments in panels b–d feature a fine-porosity glass frit.

For each electrochemical method, measurements may be conducted under isothermal or nonisothermal conditions (Figure 3). For isothermal measurements, all electrodes are heated at the same temperature. In contrast, for nonisothermal measurements, the working electrode is heated while the reference electrode is kept at ambient or colder temperature. Different electrochemical cell configurations may be used for these measurements (Figure 4) and should be chosen based on the type of data being collected and redox system investigated. For isothermal VT-CV measurements that make use of a three-electrode configuration (working, reference, and counter electrodes), a single-compartment cell or a two-compartment cell with a porous frit (or salt bridge or membrane) separating the working- and counter-electrode compartments may be utilized (Figure 4a,b). The latter should be used if the reaction at the counter electrode may interfere with the reaction happening at the working electrode. These arrangements with the working and reference electrodes in close proximity to one another minimize resistive and ohmic losses to the observed potential. Isothermal VT-OCP measurements of half-cell reactions may be carried out similarly to isothermal VT-CV measurements (Figure 4a,b). In contrast, nonisothermal VT-OCP measurements are best performed in a three-compartment electrochemical cell, where the central compartment provides a buffer region between the heated and nonheated compartments, generating a more stable temperature gradient as compared to two-compartment cells. Three-electrode and two-electrode configurations may be used to investigate half-cell and full-cell reactions, respectively (Figure 4c,d). Owing to the acquisition of cyclic voltammograms in the absence of stirring, which leads to less accurate temperature readings, and a significant internal cell resistance in three-compartment cells, we recommend against conducting nonisothermal CV measurements (vide infra). Note that these issues are not applicable to nonisothermal VT-OCP measurements as those are collected in the absence of applied current and in stirred solutions.

## Variables to Be Compensated

In addition to having a good understanding of the chemical and electrochemical properties of the studied redox system (vide supra), several variables must be thoroughly quantified and taken into consideration in efforts to give accurate and reliable values of temperature coefficients and redox reaction entropies. In this section, we will underscore key parameters that can greatly influence the variable-temperature electrochemical data collection and introduce significant errors in temperature coefficient values if not properly accounted for. We also note that the identities of the supporting electrolyte and solvent can dramatically influence the temperature coefficient values,^40^ thus comparisons between different redox-active analytes should ideally be made using identical solution conditions and measuring modes.

### Temperature

It is critical for all variable-temperature electrochemical measurements that the solution temperature is precisely monitored as close to the electrodes as possible. While hot plates, thermostatic baths, and heating units with circulating liquids provide suitable ways of heating electrochemical cells, the stated temperatures on these equipment are not necessarily representative of the true solution temperature and can only be considered estimates. The use of an internal high-resolution digital thermometer in each compartment of the electrochemical cell is strongly recommended as it provides a much more accurate temperature assessment. Such thermometer should be (i) located as physically close to the electrodes as possible and at the same height in solution (Figure 4), (ii) made of materials that are compatible with the solutions being measured, and (iii) calibrated periodically using a high-sensitivity temperature controller.

### Temperature Coefficient of the Reference Electrode

For electrochemical experiments using a three-electrode configuration, the electrochemical potential is measured against a reference electrode, such as Ag/AgNO_3_ in organic solvents and Ag/AgCl in aqueous solutions. As the reference electrode potential also varies with temperature,^19,42,43^ the temperature coefficient of the reference electrode potential must be known when relying on isothermal measurements for assessing the temperature dependence of a redox couple of interest. To illustrate, the formal potential of a redox couple at a given temperature is a summation of the measured formal potential (*E*°′_meas_) and *E*_ref_, where *E*_ref_ is the reference electrode potential. Accordingly, the temperature dependence of the formal potential of a redox couple of interest can be expressed as eq 4.
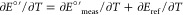
4The temperature coefficient of the reference electrode potential can, for example, be quantified through nonisothermal VT-OCP measurements between two identical reference electrodes.^16,40^ Alternatively, the temperature coefficient of the reference electrode potential may be obtained by comparing the values measured for a redox-active analyte under isothermal and nonisothermal conditions (vide infra).^42^

### Thermodiffusion

As electrochemical measurements are either performed using a high analyte concentration or in the presence of a large excess of an inert supporting electrolyte to ensure that the ionic strength of the solution is high and the electric field is homogeneous, contribution from thermodiffusion to the observed temperature coefficient may need to be considered for nonisothermal measurements when the temperature gradient across the cell leads to spatial differences in the concentrations and mobilities of ions in solution.^5,22,23,44−46^ As mentioned above, thermodiffusion contributes minimally to solution-based systems that display the thermogalvanic effect so long as the viscosity of the solution is modest. However, for highly viscous solutions and gel-based systems, the thermodiffusive contribution to the overall temperature coefficient cannot be ignored and is often larger than the thermogalvanic component (vide supra).^5,22,23,30^ In cases where thermodiffusion may be a concern, isothermal electrochemical measurements are recommended to exclusively assess the thermogalvanic effect.

### Thermal Liquid Junction Potential

A liquid junction potential develops at the interface of two electrolyte solutions if there is a difference in the concentrations and mobilities of the ions in those solutions.^42,44^ A thermal liquid junction potential is a consequence of thermodiffusion and may form at several solution–solution interfaces in electrochemical cells, such as between cell compartments in two- and three-compartment cells and near the reference electrode frit. Typical values for the temperature coefficient of the thermal liquid junction potential across a small temperature range in low-viscosity solutions are estimated to be <0.2 mV K^–1^.^42,44^ This value can be significantly higher (up to ∼0.4 mV K^–1^) for strongly acidic aqueous solutions.^46^ Notably, thermal liquid junction potentials can be minimized through the use of a salt bridge between two cell compartments held at different temperatures,^46^ as well as by employing isothermal electrochemical measurements.

### Proton Activity of Solutions

As mentioned above, the solution environment can greatly influence the temperature coefficient. Compounds that display proton-coupled electron-transfer reactions have been found to exhibit relatively high temperature coefficients due to harnessing of proton solvation entropy.^37,38^ For such redox systems, variable-temperature electrochemical measurements should be conducted at multiple pH conditions and temperature coefficients reported in specific pH ranges. Note that such systems may exhibit local pH swings near the electrode–solution interface that may be quantified using methods such as OCP transients.^47^ When redox reactions involving the generation (or consumption) of protons are studied in two- or three-compartment electrochemical cells with proton-exchange membranes (e.g., Nafion) as separators, the stability of the membranes in the desired temperature range should be separately assessed.

## Relationships between Different Variable-Temperature Electrochemical Data

In efforts to illustrate the electrochemical data obtained from different variable-temperature electrochemical measurements and the relationships between them, we collected VT-CV and VT-OCP data under isothermal and nonisothermal conditions for transition metal-based redox couples in water and acetonitrile solutions. Specifically, we chose the [Co(bipy)_3_]^3+^/[Co(bipy)_3_]^2+^ (bipy = 2,2′-bipyridine) and [Fe(CN)_6_]^3–^/[Fe(CN)_6_]^4–^ redox pairs as representative examples for nonaqueous and aqueous conditions, respectively. The results of our studies are summarized in Figures S1–S26, Tables S1–S3, and Supplementary Note in the Supporting Information.

The Co-based redox system exhibits anticipated relationships between VT-CV and VT-OCP data collected under isothermal and nonisothermal conditions. Statistically identical temperature coefficients of α_meas_ = 1.5(2)–1.7(1) mV K^–1^ were obtained for the [Co(bipy)_3_]^3+^/[Co(bipy)_3_]^2+^ redox couple of [Co(bipy)_3_](TFSI)_2_ and [Co(bipy)_3_](TFSI)_2.04_(BF_4_)_0.96_ (TFSI^–^ = bistriflimide) in acetonitrile containing 0.1 M KPF_6_ or (^*n*^Bu_4_N)(PF_6_) supporting electrolyte using VT-CV under isothermal conditions (Figures S1–S8). Furthermore, isothermal VT-OCP measurements of an equimolar solution of [Co(bipy)_3_]^2+^ and [Co(bipy)_3_]^3+^ afforded the same temperature coefficients of α_meas_ = 1.6(1)–1.7(2) mV K^–1^ (Figures S13, S15, S17, and S19). Such an agreement between isothermal VT-CV measurements of the oxidized or reduced form of a redox-active compound and isothermal VT-OCP analysis of an equimolar mixture of both forms has been reported for other redox couples.^16^ Analogous VT-OCP measurements conducted under nonisothermal conditions yielded temperature coefficients of α = 2.1(2)–2.2(1) mV K^–1^ (Figures S14, S16, S18, and S20). These results are in a good agreement with eq 4, as the temperature coefficients for the Ag/AgNO_3_ reference electrode potential in acetonitrile solutions containing 0.1 M KPF_6_ or (^*n*^Bu_4_N)(PF_6_) are 0.48(7) and 0.43(6) mV K^–1^, respectively. Together, these studies (Tables S1 and S2) underscore that true temperature coefficients may be obtained using either direct nonisothermal VT-OCP measurements or isothermal measurements (VT-CV or VT-OCP) after correcting for the temperature sensitivity of the reference electrode potential (eq 4).

Similar to the Co-based redox system, VT-OCP measurements of the [Fe(CN)_6_]^3–^/[Fe(CN)_6_]^4–^ redox couple reveal statistically identical temperature coefficients of α = −1.4(2) and −1.4(1) mV K^–1^ in water containing 0.1 M KNO_3_ under nonisothermal and isothermal conditions, respectively, after correcting for the temperature sensitivity of the Ag/AgCl reference electrode potential for the latter (Figures S21–S24 and Table S3). In contrast, isothermal VT-CV measurements of solutions of [Fe(CN)_6_]^4–^ or [Fe(CN)_6_]^3–^ afforded significantly different results (Figures S9–S12 and Table S3), with only the data collected for [Fe(CN)_6_]^3–^ being similar to the VT-OCP data (Table S3). The less negative temperature coefficient obtained from VT-CV measurements of [Fe(CN)_6_]^4–^ likely arises from the difference in hydration and ionic interactions between the two redox forms of the Fe-based redox system.^1,22,40^ We further note that the nature of the Ag/AgCl reference electrode impacts variable-temperature electrochemical measurements conducted under isothermal conditions (Figures S25 and S26, and Experimental Section). Accordingly, care must be taken in the choice of supporting electrolyte and reference electrode for variable-temperature electrochemical analysis.

One key difference between nonisothermal VT-OCP and isothermal VT-CV methods is that the use of the former method for homogeneous liquid-phase redox reactions necessitates the chemical isolation of both the oxidized and reduced forms of the redox couple of interest. Another important distinction is that multiple redox couples of a given redox-active compound may be simultaneously analyzed using isothermal VT-CV.^16,48^ With this in mind, we refer researchers to nonisothermal VT-OCP measurements if a single redox event of chemically isolable species is of interest, to avoid introduction of error to the temperature coefficient from assessment of the temperature coefficient of the reference electrode potential. On the other hand, researchers interested in multiple redox events for a given analyte are directed to isothermal VT-CV measurements, and encouraged to choose a reference electrode and solution conditions for which the temperature coefficient of the reference electrode potential is known. Certainly, performing both types of measurements would be best to check for consistency, as reported herein. For multiphase redox processes and kinetically sluggish reactions, we recommend VT-OCP measurements in efforts to achieve steady-state conditions and minimize the influence of kinetics as no current flows through the system and longer measurement times are possible.

## Conclusions and Summary of Recommended Best Practices

Considering our results and other reported variable-temperature electrochemical studies on solution-phase redox systems (see Table S4 for examples), we suggest the following recommendations for researchers for acquiring and reporting variable-temperature electrochemical data in efforts to increase accuracy and standardize protocols in the field.

- *Identify the Properties of the Desired Redox System*. As a first step, employ analytical and electroanalytical tools to decipher the chemical and electrochemical properties of the redox system of interest. This includes assessment of chemical equilibria, thermal and solution stability, electrochemical stability, and kinetic stability. The choice of method to quantify temperature coefficients will be significantly impacted by the characteristics of the given redox system.
- *Carefully Consider Experimental Protocols*. Provide a detailed experimental section describing the conditions used for electrochemical measurements, including the type of measurements, compositions of solutions, experimental setup, and what (if any) corrections are made to the data. Be aware of experimental conditions where additional corrections, such as for thermodiffusion and thermal liquid junction potential, should be made. Use the same experimental conditions (solvent, supporting electrolyte, measuring mode, etc.) when comparing temperature coefficients of different redox-active analytes.
- *Record Temperature with Accuracy*. Use an internal high-resolution digital thermometer to monitor the temperature in the vicinity of the electrodes to ensure accurate and reliable temperature measurements. The thermometers should be placed close to the electrodes in solutions and calibrated periodically using an external temperature controller.
- *Provide Appropriate Quantification of Temperature Coefficients*. Use VT-OCP rather than VT-CV measurements for electrochemical systems in which both the oxidized and reduced forms of the redox-active analyte can be chemically isolated. The VT-OCP method can also yield accurate temperature coefficients in cases where sluggish kinetics of electron-transfer reactions may be a concern. Carry out VT-CV measurements under isothermal conditions only, as nonisothermal VT-CV measurements are typically less reliable. Correct all variable-temperature electrochemical data collected under isothermal conditions for the temperature coefficient of the reference electrode potential, determined using the same reference electrode and supporting electrolyte solution as in the presence of the redox-active analyte. For systems that are sensitive to solution proton activity, report temperature coefficients for specific pH ranges.

To conclude, we hope that this Viewpoint provides guidance to the electrochemistry research community, inspires more researchers to engage in variable-temperature electrochemical studies, and ultimately stimulates new research directions relying on variable-temperature electrochemistry.

## Supporting Information Available

Experimental details, additional electrochemical data, and overview of select literature reports on variable-temperature electrochemical studies of solution-phase systems (PDF)
