# Supplementary material for: Best Practices for Variable-Temperature Electrochemistry Experiments and Data Reporting
Source: ACS Energy Lett. 2025 Mar 7;10(4):1542–9. doi: 10.1021/acsenergylett.5c00308 (PMC11998081; doi:10.1021/acsenergylett.5c00308)
Supplement: Supplementary file 1 — nz5c00308_si_001.pdf [file nz5c00308_si_001.pdf]

Supporting Information for:

Best Practices for Variable-Temperature Electrochemistry Experiments and Data Reporting

Anyesh De, Mamta Dagar, Bryce Kneer, James Kim, and *Agnes E. Thorarinsdottir**
Department of Chemistry, University of Rochester, Rochester, New York 14627, USA

*Correspondence to: agnes.thorarinsdottir@rochester.edu (A.E.T.)

Table of Contents

A. Experimental Section

General Considerations……………………………..………………………………………….S3
Synthesis of $[Co(bipy)_3](TFSI)_2$…………………...……………………………………… S3
Synthesis of $[Co(bipy)_3](TFSI)_{2.04}(BF_4)_{0.96}$ …………………………………………….... S3
Electrochemical Measurements……………………………………………………………..S4
Other Physical Measurements..……………………………………………………………...S7
Electrochemical Equations…………………………………………………………………...S7

B. Supplementary Note

Incompatibility of $K_3[Fe(CN)_6]$ with KCl in water……………………………………………... S8

C. Supplementary Figures

Figure S1. Isothermal VT-CV data of $[Co(bipy)_3]^{2+}$ in MeCN with KPF_6..……………………...S9
Figure S2. Isothermal VT-CV data of $[Co(bipy)_3]^{2+}$ in MeCN with $(^nBu_4N)(PF_6)$...…………...S10
Figure S3. Isothermal VT-CV data of $[Co(bipy)_3]^{3+}$ in MeCN with KPF_6..…………………...S11
Figure S4. Isothermal VT-CV data of $[Co(bipy)_3]^{3+}$ in MeCN with $(^nBu_4N)(PF_6)$...…………...S12
Figure S5. Plot of $E_{1/2}$ vs T from VT-CV data of $[Co(bipy)_3]^{2+}$ in MeCN with KPF_6…………S13
Figure S6. Plot of $E_{1/2}$ vs T from VT-CV data of $[Co(bipy)_3]^{2+}$ in MeCN with $(^nBu_4N)(PF_6)$...S14
Figure S7. Plot of $E_{1/2}$ vs T from VT-CV data of $[Co(bipy)_3]^{3+}$ in MeCN with KPF_6…………S15
Figure S8. Plot of $E_{1/2}$ vs T from VT-CV data of $[Co(bipy)_3]^{3+}$ in MeCN with $(^nBu_4N)(PF_6)$...S16
Figure S9. Isothermal VT-CV data of $[Fe(CN)_6]^{4-}$ in water with KNO_3…..…………………...S17
Figure S10. Isothermal VT-CV data of $[Fe(CN)_6]^{3-}$ in water with KNO_3…..……………………S18
Figure S11. Plot of $E_{1/2}$ vs T from VT-CV data of $[Fe(CN)_6]^{4-}$ in water with KNO_3…..………S19
Figure S12. Plot of $E_{1/2}$ vs T from VT-CV data of $[Fe(CN)_6]^{3-}$ in water with KNO_3…..………S20
Figure S13. Isothermal VT-OCP data of $[Co(bipy)_3]^{3+/2+}$ in MeCN with KPF_6………………S21

Figure S14. Nonisothermal VT-OCP data of $[Co(bipy)_3]^{3+/2+}$ in MeCN with KPF_6…………S22
Figure S15. Isothermal VT-OCP data of $[Co(bipy)_3]^{3+/2+}$ in MeCN with $(^nBu_4N)(PF_6)$………S23
Figure S16. Nonisothermal VT-OCP data of $[Co(bipy)_3]^{3+/2+}$ in MeCN with $(^nBu_4N)(PF_6)$…S24
Figure S17. Plot of E_{OCP} vs T for $[Co(bipy)_3]^{3+/2+}$ in MeCN with KPF_6………………………....S25
Figure S18. Plot of E_{OCP} vs ΔT for $[Co(bipy)_3]^{3+/2+}$ in MeCN with KPF_6……………………....S26
Figure S19. Plot of E_{OCP} vs T for $[Co(bipy)_3]^{3+/2+}$ in MeCN with $(^nBu_4N)(PF_6)$……………....S27
Figure S20. Plot of E_{OCP} vs ΔT for $[Co(bipy)_3]^{3+/2+}$ in MeCN with $(^nBu_4N)(PF_6)$……………...S28
Figure S21. Isothermal VT-OCP data of $[Fe(CN)_6]^{3-/4-}$ in water with KNO_3………………...S29
Figure S22. Nonisothermal VT-OCP data of $[Fe(CN)_6]^{3-/4-}$ in water with KNO_3……………S30
Figure S23. Plot of E_{OCP} vs T for $[Fe(CN)_6]^{3-/4-}$ in water with KNO_3…………………………S31
Figure S24. Plot of E_{OCP} vs ΔT for $[Fe(CN)_6]^{3-/4-}$ in water with KNO_3…………………………S32
Figure S25. Nonisothermal VT-OCP data of $[Fe(CN)_6]^{3-/4-}$ in water with KNO_3 w/LF-RE…S33
Figure S26. Plot of E_{OCP} vs ΔT for $[Fe(CN)_6]^{3-/4-}$ in water with KNO_3 w/LF-RE………………S34

D. Supplementary Tables

Table S1. Summary of VT electrochemistry for $[Co(bipy)_3]^{3+/2+}$ in MeCN with KPF_6………..S35
Table S2. Summary of VT electrochemistry for $[Co(bipy)_3]^{3+/2+}$ in MeCN with $(^nBu_4N)(PF_6)$..S36
Table S3. Summary of VT electrochemistry for $[Fe(CN)_6]^{3-/4-}$ in water with KNO_3…………S37
Table S4. Summary of select VT electrochemical studies from the literature………………S38

E. References……………………………………………………………………………….. S40

A. Experimental Section

General Considerations. Unless otherwise specified, the manipulations described below were carried out under ambient atmosphere and temperature. Air- and water-free manipulations were performed under a dry dinitrogen atmosphere in a MBraun UNIlab pro glovebox. Glassware was oven-dried at 125 °C for at least 4 h and allowed to cool in an evacuated antechamber prior to use in the glovebox. Acetonitrile (MeCN) used inside the glovebox and for electrochemical experiments was dried using a commercial solvent purification system from Pure Process Technology and stored over 3 Å molecular sieves (Supelco, Inc.) prior to use. Diethyl ether (Et_2O) and methanol (MeOH) solvents used outside the glovebox were purchased from Fisher Scientific. Ultrapure water (18.2 MΩ cm) was obtained from an Arium Mini water purification system from Sartorius. Deuterated dimethyl sulfoxide was purchased from Cambridge Isotope Laboratories, Inc. ($^{n}Bu_4N$)(PF_6) used for electrochemical measurements was purchased from A2B Chem LLC and recrystallized two times from ethanol and dried under vacuum for at least 12 h prior to use. All other reagents, including $K_4[Fe(CN)_6]\cdot 3H_2O$, $K_3[Fe(CN)_6]$, KPF_6, and KNO_3, were purchased from commercial vendors and used without further purification.

Synthesis of [Co(bipy)$_3$](TFSI)$_2$. This compound was synthesized following a modified literature procedure.[1] Cobalt(II) chloride hexahydrate ($CoCl_2\cdot 6H_2O$; 0.98 g, 4.12 mmol) and 2,2'-bipyridine (bipy; 2.22 g, 14.21 mmol) were dissolved in MeOH (25 mL) to give a pink solution. This solution was heated to reflux and left stirring at that temperature for 2 h. Afterwards, the pink solution was cooled to 25 °C and a colorless solution of lithium bis(trifluoromethanesulfonyl)imide (LiTFSI; 2.87 g, 10.00 mmol) in MeOH (5 mL) was added. The resulting solution was stirred at 25 °C for 3 h and the MeOH solvent was slowly evaporated in the fume hood to give orange brown needle-shaped crystals within 16 h. These crystals were collected by vacuum filtration and washed with ultrapure water (3 × 50 mL), followed by Et_2O (10 mL), and dried under suction on the filter for 20 min. Further drying under vacuum for 8 h afforded the title compound as an orange brown crystalline solid (4.14 g, 92%). Anal. Calcd. for $C_{34}H_{24}CoF_{12}N_8O_8S_4$: C, 37.54; H, 2.22; N, 10.30%. Found: C, 37.40; H, 2.27; N, 10.30%. ^1H NMR (400 MHz, $(CD_3)_2SO$, 22 °C): δ 8.66 (d, 6H), 8.37 (d, 6H), 7.92 (t, 6H), 7.43 (t, 6H).

Synthesis of [Co(bipy)$_3$](TFSI)$_{2.04}$(BF$_4$)$_{0.96}$. Inside the glovebox, [Co(bipy)$_3$](TFSI)$_2$ (2.35 g, 2.16 mmol) was dissolved in dry MeCN (15 mL) to give a dark orange solution. To this stirring solution, a colorless suspension of nitrosonium tetrafluoroborate ($NOBF_4$, 0.32 g, 2.74 mmol) in dry MeCN

(5 mL) was added and the resulting light orange solution was stirred at 25 °C for 1.5 h. Afterwards, the reaction was brought outside the glovebox and a colorless solution of LiTFSI (3.10 g, 10.80 mmol) in dry MeCN (15 mL) was added and the solution was stirred at 25 °C for additional 1.5 h. The light orange solution was then concentrated under reduced pressure and the resulting orange oil was triturated with MeOH (50 mL), first at 25 °C and then at −35 °C for 2 h, to induce the precipitation of the product as a yellow solid. This yellow solid was collected by vacuum filtration, washed with cold MeOH (50 mL), Et_2O (10 mL), ultrapure water (100 mL), and Et_2O (5 mL) in this order. Recrystallization from MeOH, followed by washing with Et_2O (20 mL) and drying under vacuum for 4 h afforded the title compound as a yellow solid (1.52 g, 60%). Anal. Calcd. for $C_{34.08}H_{24}CoB_{0.96}F_{16.08}N_{8.04}O_{8.16}S_{4.08}$: C, 34.62; H, 2.05; N, 9.52%. Found: C, 34.63; H, 1.95; N, 9.32%. ^{1}H NMR (400 MHz, $(CD_3)_2SO$, 22 °C): δ 9.03 (d, 6H), 8.60 (t, 6H), 7.80 (t, 6H), 7.44 (d, 6H). $^{19}F\{^{1}H\}$ NMR (376 MHz, $(CD_3)_2SO$, 22 °C): δ −78.60, −148.09.

Electrochemical Measurements. All electrochemical experiments were performed in electrochemical glass cells using deoxygenated solvents under argon purging using CH Instruments 760E electrochemical workstation. Isothermal and nonisothermal measurements were carried out using single-compartment and three-compartment (custom-made with fine frits separating the two side compartments from the middle compartment, Adam & Chittenden Scientific) glass cells, respectively. For nonisothermal measurements, the working electrode was placed in one of the side compartments that was heated during the measurement, while the reference and counter electrodes were placed in the other side compartment that was held at ambient temperature. The middle compartment was filled with the same solution as the side compartments. All glass cells were washed with concentrated nitric acid (ACS Plus, 15.8 N, Fisher Scientific) followed by ultrapure water and oven-dried at 125 °C for at least 1 h prior to use. Teflon-coated magnetic stir bars (Fisher Scientific) were washed with aqua regia followed by ultrapure water and oven-dried at 125 °C for at least 4 h prior to use. The temperature of the electrochemical cells was controlled using a hot plate. The actual temperature near the working electrode was monitored using a thermocouple (stainless steel), which was calibrated using an external temperature controller (Omega Engineering, CS8DPT). The $[Co(bipy)_3]^{3+}/[Co(bipy)_3]^{2+}$ redox couple was analyzed in dry MeCN containing 0.1 M KPF_6 or $(^{n}Bu_4N)(PF_6)$ supporting electrolyte using glassy carbon (3-mm diameter, Pine Research Instrumentation, Inc.) working electrode, a non-aqueous $Ag/AgNO_3$ reference electrode (CH Instruments, Inc.) filled with a dry MeCN solution containing 0.01 M $AgNO_3$ and 0.1 M $(^{n}Bu_4N)(PF_6)$, and a counter electrode composed of a platinum mesh (99.9%, 52 mesh woven from 0.1-mm-diameter wire, Fisher Scientific) attached

to a platinum wire (99.95%, 0.5-mm diameter, Fisher Scientific). The $[Fe(CN)_6]^{3-}$/$[Fe(CN)_6]^{4-}$ redox couple was analyzed in deoxygenated ultrapure water containing 0.1 M KNO_3 supporting electrolyte using Pt (3-mm diameter, BASi) working electrode, a glass Ag/AgCl (filled with a 3 M aqueous KCl solution, BASi) or a leak-free Ag/AgCl reference electrode (Innovative Instruments, Inc.), and an analogous Pt-based counter electrode as used for the Co-based system. Before use, working electrodes were polished using alumina powder (0.05 μm, Allied High Tech Products, Inc.) on a microfiber polishing cloth and platinum counter electrodes were cleaned by soaking in concentrated nitric acid followed by drying with a butane flame.

Cyclic voltammetry (CV) measurements were conducted under isothermal conditions using positive scan direction at a scan rate of 100 mV s^{-1} in the potential window of 0.8 V to −0.4 V. All potentials are referenced to the respective reference electrodes, Ag/$AgNO_3$ in MeCN and Ag/AgCl in water. Samples for measurements were prepared by dissolving either the oxidized or reduced form of each redox-active analyte in ~10 mL of a 0.1 M electrolyte solution (KPF_6 or ($^nBu_4N$)(PF_6) in MeCN for the Co-based system and KNO_3 in water for the Fe-based system) to give an analyte concentration of 2.5 mM. Uncompensated solution resistance (R_u) was determined using the potential step method (amplitude of 50 mV) around the open circuit potential (OCP; E_{OCP}). Values of ~50–90 Ω and ~60–120 Ω were obtained for experiments conducted in MeCN and water (using glass Ag/AgCl reference electrode filled with a 3 M aqueous KCl solution), respectively. Note, however, that all potentials are reported without applying iR_u compensation as identical CV traces were obtained with and without applying such compensation. Negative currents correspond to anodic reactions (oxidation) and positive currents correspond to cathodic reactions (reduction). The temperature of the cell was increased in 3–5 °C intervals in the temperature range 20–45 °C and monitored near the working and reference electrodes using a previously calibrated thermocouple. The cell was maintained at a fixed temperature while recording the CV data. Solutions were stirred between measurements at different temperatures but paused during data recording to reduce disturbance at the working electrode for more accurate measurements. Half-cell potentials ($E_{1/2}$) for each redox couple were extracted using the anodic and cathodic peak potentials from the variable-temperature CV (VT-CV) data and plotted against temperature. As the diffusion coefficients for the oxidized and reduced forms of the studied redox couples are expected to be similar,[2] we make the estimation that the formal potential and half-wave potential are equal ($E^{0\prime} \approx E_{1/2}$), thus the slopes of the linear fits to the data of $E_{1/2}$ vs temperature plots afford the temperature coefficients (α) of the redox couples with respect to the reference electrode potential (vide infra; eq S1). Notably, the electrochemical potentials of the Ag/$AgNO_3$ and Ag/AgCl reference electrodes are also sensitive to temperature.[3–

[5] Accordingly, the true temperature coefficient of a given redox couple was obtained after correcting for the temperature coefficient of the respective reference electrode potential (vide infra, eqs S2 and S3). The temperature coefficients of the reference electrode potentials were assessed following a previously published protocol.[6] Values of 0.48(7) mV K^{-1} and 0.43(6) mV K^{-1} were obtained for Ag/$AgNO_3$ in MeCN containing 0.1 M KPF_6 or ($^n Bu_4N$)(PF_6), respectively, and a value of 0.2(1) mV K^{-1} was obtained for glass Ag/AgCl (filled with a 3 M aqueous KCl solution) in water containing 0.1 M KNO_3. These values are in good agreement with literature values.[3–6] Note that measurements of the temperature coefficient of the electrochemical potential of leak-free Ag/AgCl reference electrodes in water containing 0.1 M KNO_3 were not reproducible, thus we only used leak-free Ag/AgCl reference electrodes for nonisothermal OCP experiments and relied on glass Ag/AgCl reference electrodes for the full set of experiments.

OCP measurements were carried out under isothermal and nonisothermal conditions using an equimolar (2.5 mM of each charge state) ratio of the oxidized and reduced form of each redox-active analyte in the same electrolyte solutions as used for the CV measurements. The total volume of the electrolyte solution was ~10 mL and ~22 mL for isothermal and nonisothermal experiments, respectively. For the nonisothermal experiments, ~10 mL of solution was added to each of the side compartments and the remaining ~2 mL was added to the middle compartment to fully cover the fritted separators. The temperature of the cell was increased in 2–4 °C intervals in the temperature range 20–45 °C and monitored near the working and reference electrodes using a previously calibrated thermocouple. The cell was maintained at a fixed temperature while recording the OCP data. Solutions were stirred during and between measurements at different temperatures. At each temperature, E_{OCP} was recorded for 60 s. The average E_{OCP} values were plotted against temperature (isothermal) or temperature difference (nonisothermal), and the slopes of the linear fits to the data of E_{OCP} vs temperature plots or E_{OCP} vs temperature difference plots afforded the temperature coefficients of the redox couples. For isothermal OCP measurements, the obtained temperature coefficients were corrected for the temperature sensitivity of the reference electrode potentials as described above for isothermal VT-CV measurements. However, for nonisothermal OCP measurements, the obtained temperature coefficients represent the true values for given redox couples.

Note that all temperature coefficients are reported in units of mV K^{-1}, as is the convention in the field. The temperature coefficient values (after correction if applicable) were used to estimate the redox reaction entropies (ΔS_{redox}; eq S4). Independently prepared samples of each type were measured at least three times to ensure reproducibility and error bars denote standard deviations of those measurements. The provided data are representative examples.

Other Physical Measurements. All ^{1}H and $^{19}F\{^{1}H\}$ NMR spectra were collected at 22 °C at 400 MHz and 376 MHz frequencies, respectively, on Bruker 400 MHz (9.4 T) or automated JEOL 400 MHz (9.4 T) spectrometers. All chemical shift values (δ) are reported in ppm. ^{1}H NMR spectra are referenced to residual proton signals from the deuterated solvent (2.50 ppm for $(CD_3)_2SO$). $^{19}F\{^{1}H\}$ NMR spectra are referenced to an external standard of $CFCl_3$ (δ = 0 ppm). The MestReNova 10.0 NMR data processing software was used to analyze and process all recorded NMR spectra. Elemental analysis was performed at the CENTC Elemental Analysis Facility at the University of Rochester. Samples for analysis were weighed with a PerkinElmer Model AD 6000 Autobalance and their compositions were determined with a PerkinElmer 2400 Series II Elemental Analyzer. Air-sensitive samples were handled in a VAC Atmospheres glovebox.

Electrochemical Equations. The temperature coefficient of a given redox couple is given by eq S1.

$$\frac{\partial E^{0\prime}}{\partial T} \approx \frac{\partial E_{1/2}}{\partial T} = \alpha \qquad \text{(S1)}$$

In this equation, $E^{0\prime}$ and $E_{1/2}$ denote the formal potential and half-wave potential, respectively, T denotes the temperature, and α is the temperature coefficient with respect to the given reference electrode potential.

As the reference electrode potentials are sensitive to temperature, variable-temperature electrochemical measurements conducted under isothermal conditions require corrections as illustrated in eqs S2 and S3.

$$\frac{\partial E_{1/2}}{\partial T} = \frac{\partial E_{1/2\text{-meas}}}{\partial T} + \frac{\partial E_{\text{ref}}}{\partial T} \qquad \text{(S2)}$$

$$\alpha = \alpha_{\text{meas}} + \alpha_{\text{ref}} \qquad \text{(S3)}$$

In these equations, $E_{1/2\text{-meas}}$ is the measured half-wave potential, E_{ref} is the potential of the reference electrode, α is the true temperature coefficient, α_{meas} is the measured temperature coefficient, and α_{ref} is the temperature coefficient of the reference electrode potential.

The temperature coefficient values for the redox couples may be used to estimate the associated redox reaction entropies (ΔS_{redox}) using eq S4.

$$\Delta S_{\text{redox}} = nF\alpha \qquad \text{(S4)}$$

In this equation, n is the number of electrons transferred in the given reaction, F is Faraday's constant, and α is the temperature coefficient (after correction if applicable).

B. Supplementary Note

For electrochemical measurements of the $[Fe(CN)_6]^{3-}/[Fe(CN)_6]^{4-}$ redox couple in aqueous media, KCl was initially chosen as a supporting electrolyte, however, we found that the yellow solution of 2.5 mM $K_3[Fe(CN)_6]$ and 0.1 M KCl in ultrapure water turned green blue within 10 min at 20 °C. Upon further experimentation, it was observed that a blue residue was generated at the surface of the stainless-steel thermocouple used for temperature measurements. The blue residue and the associated green blue color of the solution became more pronounced as time passed, as illustrated in the series of pictures displayed below (time progresses from left to right, with the top panel preceding the bottom panel). Similar observations were made upon replacing KCl with NaCl or LiCl as supporting electrolytes. In contrast, using other potassium salts like KNO_3 or KPF_6 in place of KCl did not result in the formation of a blue residue. Hence, we postulate that this precipitation arises from a chemical reaction between Cl^- and the stainless-steel material of the thermocouple. Interestingly, no blue residue was observed when $K_3[Fe(CN)_6]$ was replaced with $K_4[Fe(CN)_6]$. The exact nature of the chemical reaction occurring is still unclear and under further investigation in our laboratory. For these reasons, we used KNO_3 as the supporting electrolyte for electrochemical measurements of the $[Fe(CN)_6]^{3-}/[Fe(CN)_6]^{4-}$ redox couple in water solutions.

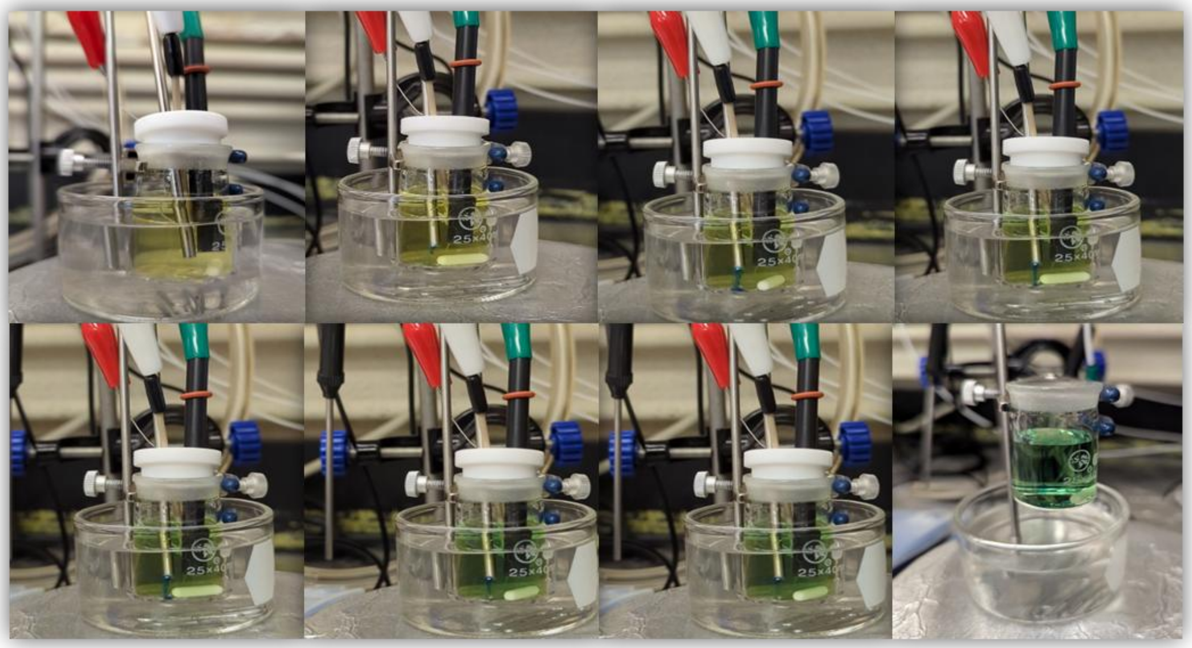

C. Supplementary Figures

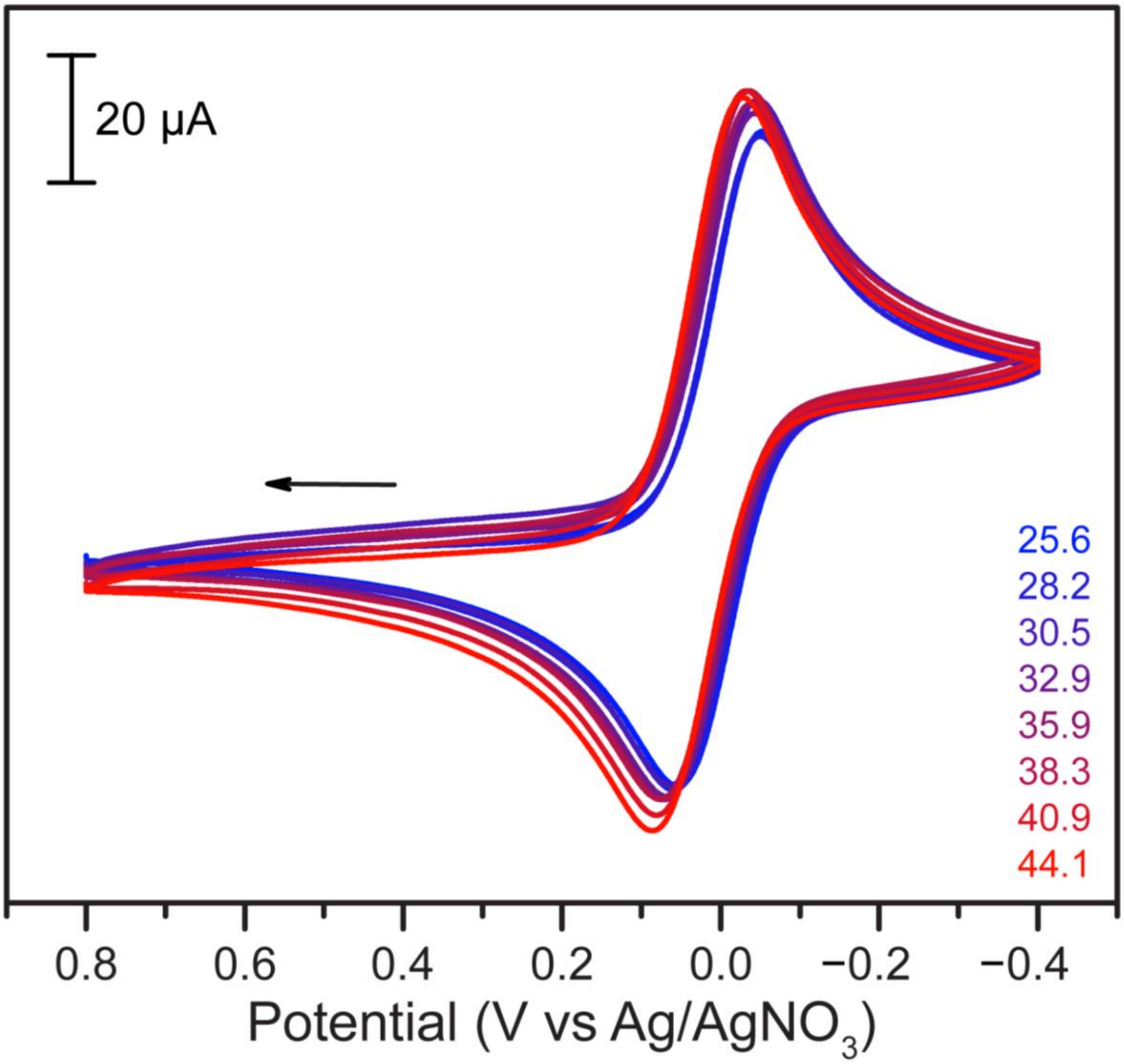

Figure S1. Representative example of VT-CV data collected under isothermal conditions for 2.5 mM of $[Co(bipy)_3]^{2+}$ in MeCN containing 0.1 M KPF_6 supporting electrolyte using 100 mV s^{-1} scan rate. The black arrow denotes the scan direction, and the colored numbers denote the solution temperatures for respective voltammograms in °C. Glassy carbon, $Ag/AgNO_3$, and Pt mesh were used as working, reference, and counter electrodes, respectively.

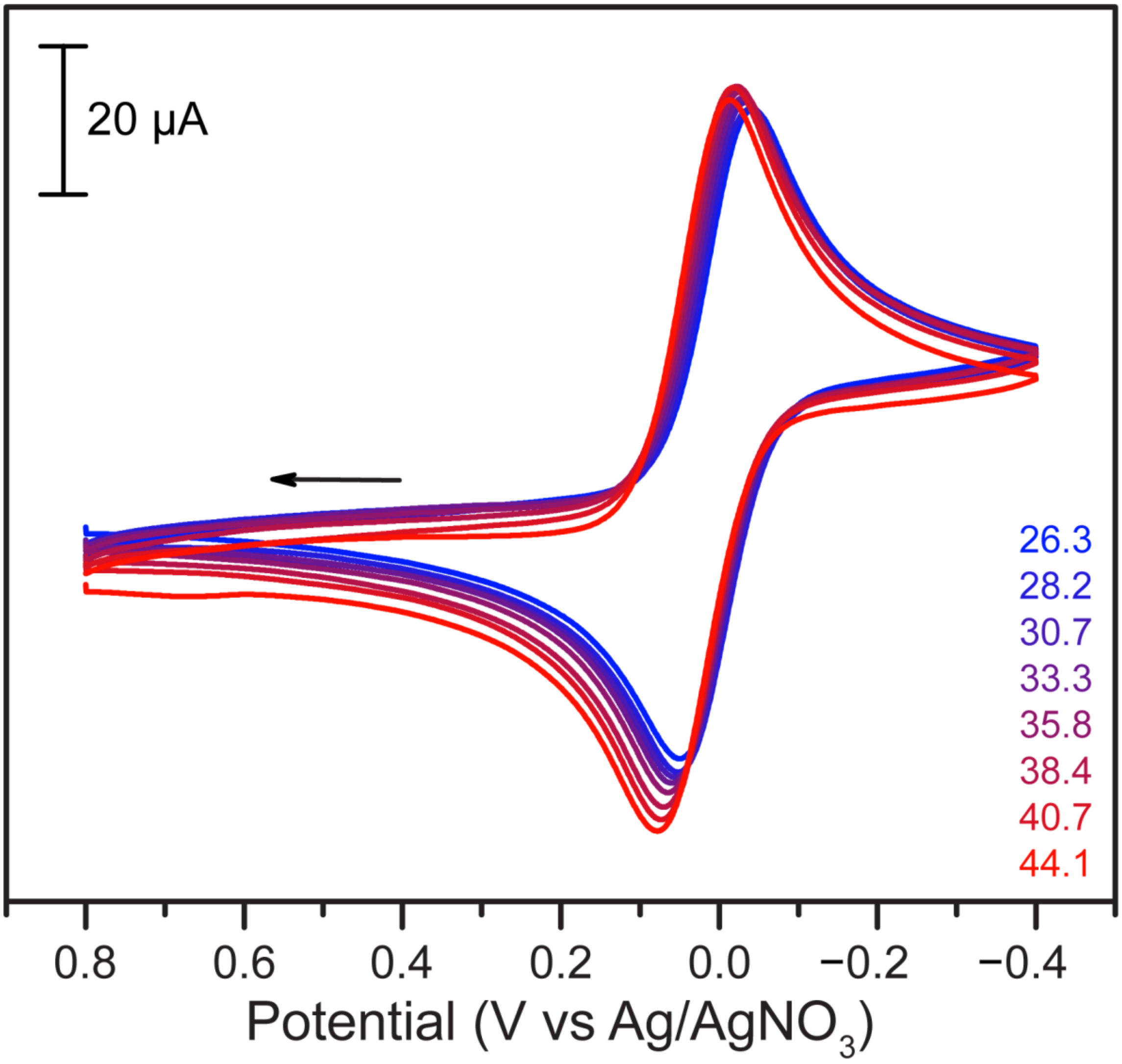

Figure S2. Representative example of VT-CV data collected under isothermal conditions for 2.5 mM of $[Co(bipy)_3]^{2+}$ in MeCN containing 0.1 M ($^{n}Bu_4N$)(PF_6) supporting electrolyte using 100 mV s^{-1} scan rate. The black arrow denotes the scan direction, and the colored numbers denote the solution temperatures for respective voltammograms in °C. Glassy carbon, Ag/$AgNO_3$, and Pt mesh were used as working, reference, and counter electrodes, respectively.

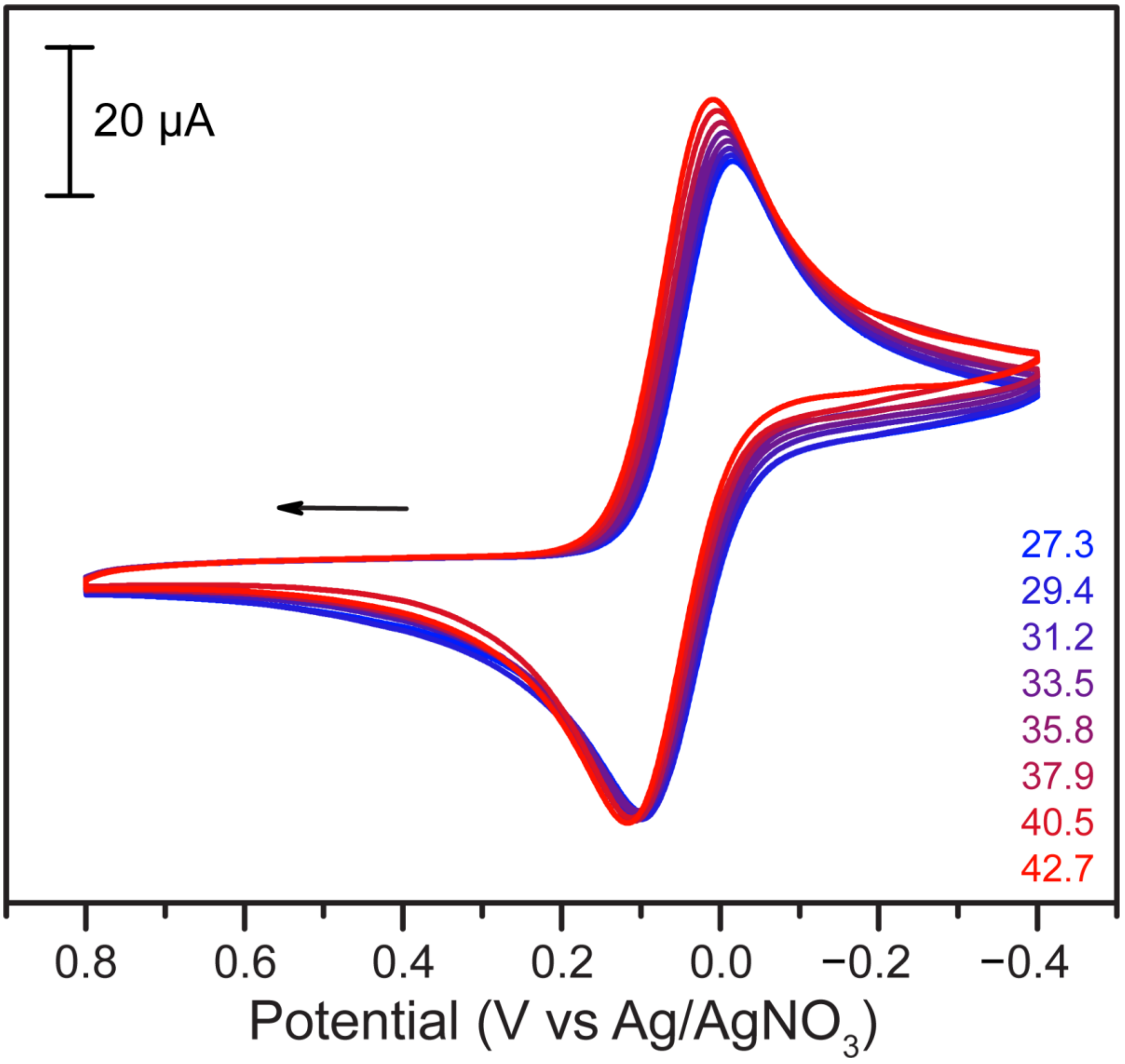

Figure S3. Representative example of VT-CV data collected under isothermal conditions for 2.5 mM of $[Co(bipy)_3]^{3+}$ in MeCN containing 0.1 M KPF_6 supporting electrolyte using 100 mV s^{-1} scan rate. The black arrow denotes the scan direction, and the colored numbers denote the solution temperatures for respective voltammograms in °C. Glassy carbon, $Ag/AgNO_3$, and Pt mesh were used as working, reference, and counter electrodes, respectively.

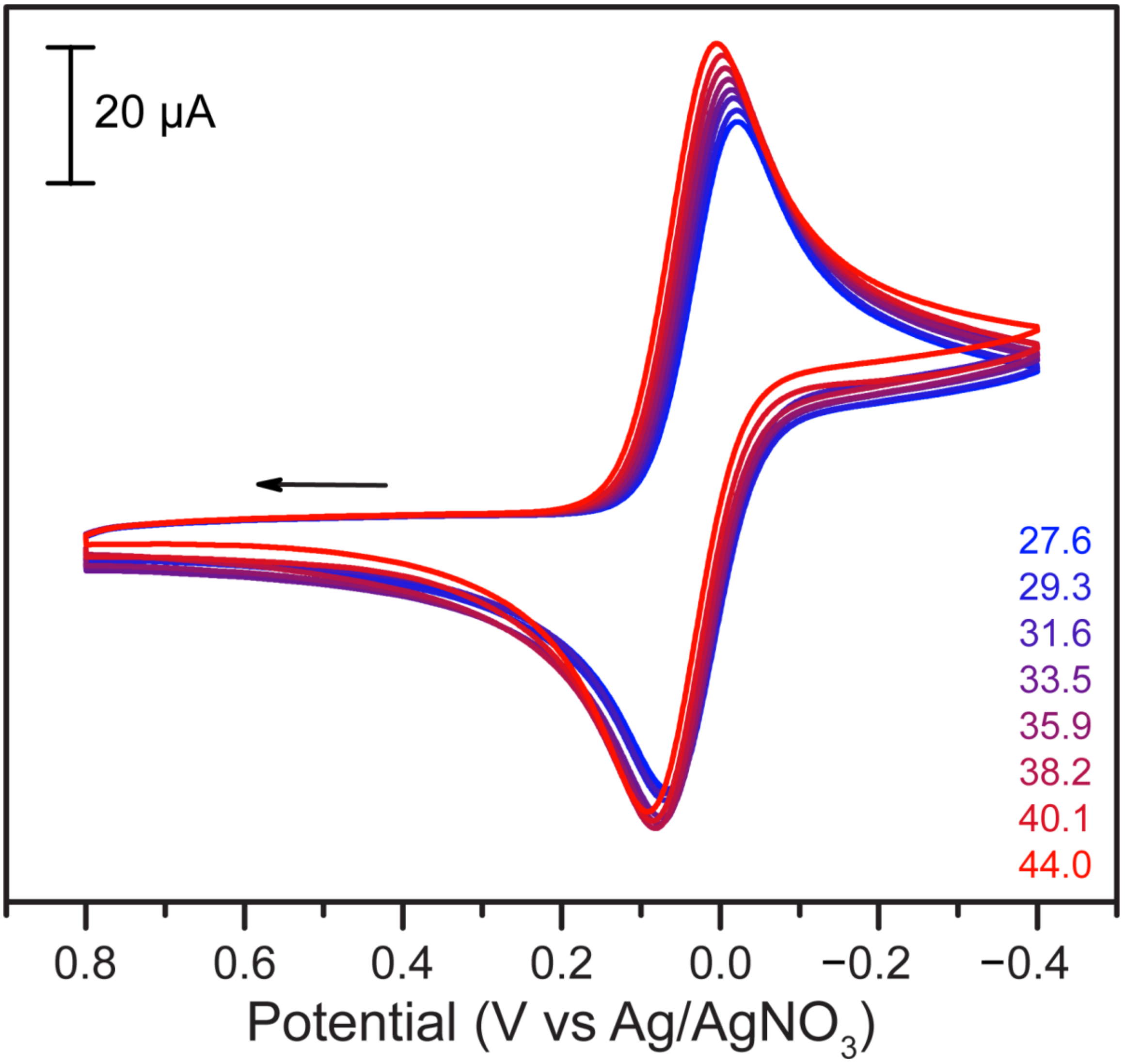

Figure S4. Representative example of VT-CV data collected under isothermal conditions for 2.5 mM of $[Co(bipy)_3]^{3+}$ in MeCN containing 0.1 M ($^n Bu_4N$)(PF_6) supporting electrolyte using 100 mV s^{-1} scan rate. The black arrow denotes the scan direction, and the colored numbers denote the solution temperatures for respective voltammograms in °C. Glassy carbon, Ag/$AgNO_3$, and Pt mesh were used as working, reference, and counter electrodes, respectively.

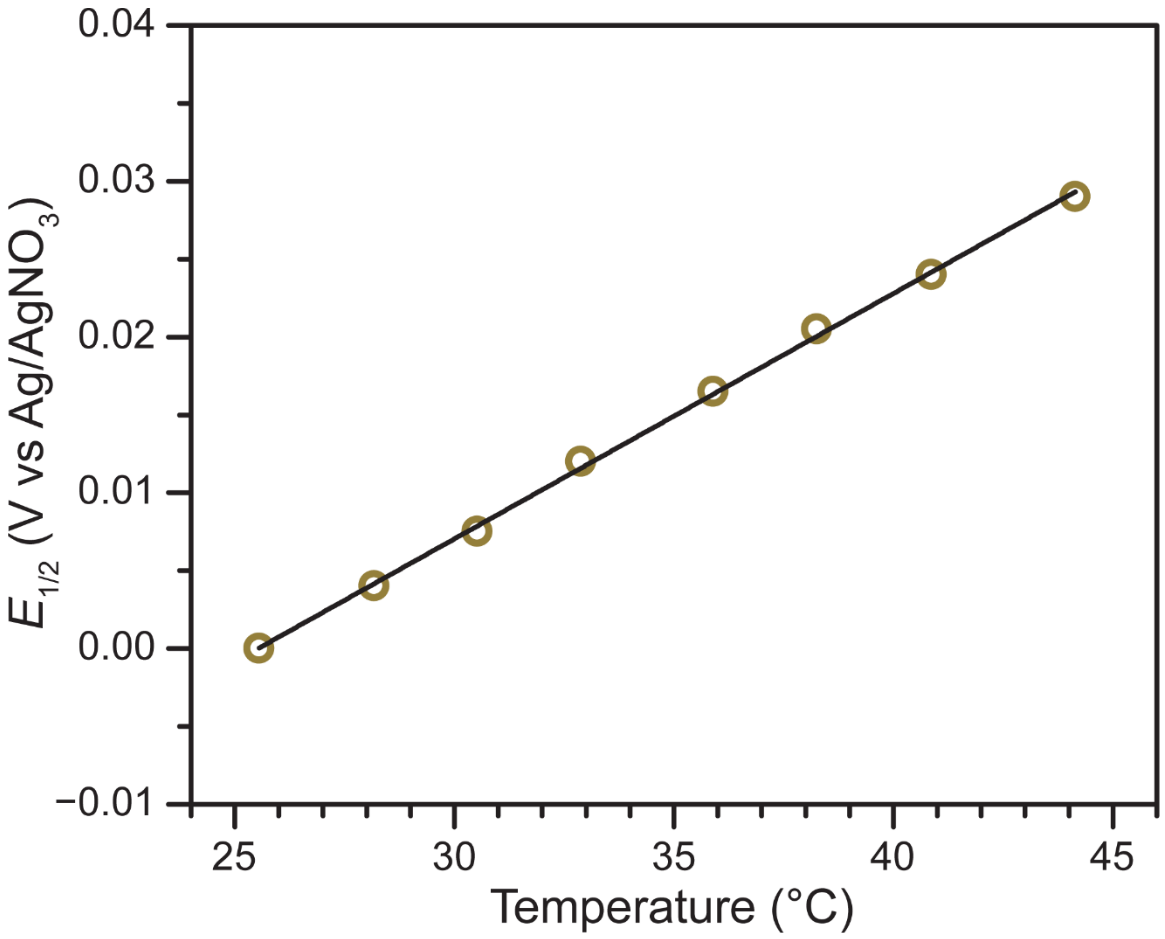

Figure S5. Representative example of a plot of $E_{1/2}$ vs temperature obtained from VT-CV data displayed in Figure S1. The data were collected under isothermal conditions for 2.5 mM of $[Co(bipy)_3]^{2+}$ in MeCN containing 0.1 M KPF_6 supporting electrolyte using 100 mV s^{-1} scan rate. Gold circles denote experimental data, and the black line corresponds to a linear fit to the data. The slope of the linear fit to the data represents the temperature coefficient for the $[Co(bipy)_3]^{3+}/[Co(bipy)_3]^{2+}$ redox couple as compared to the $Ag/AgNO_3$ reference electrode potential in the given solution conditions.

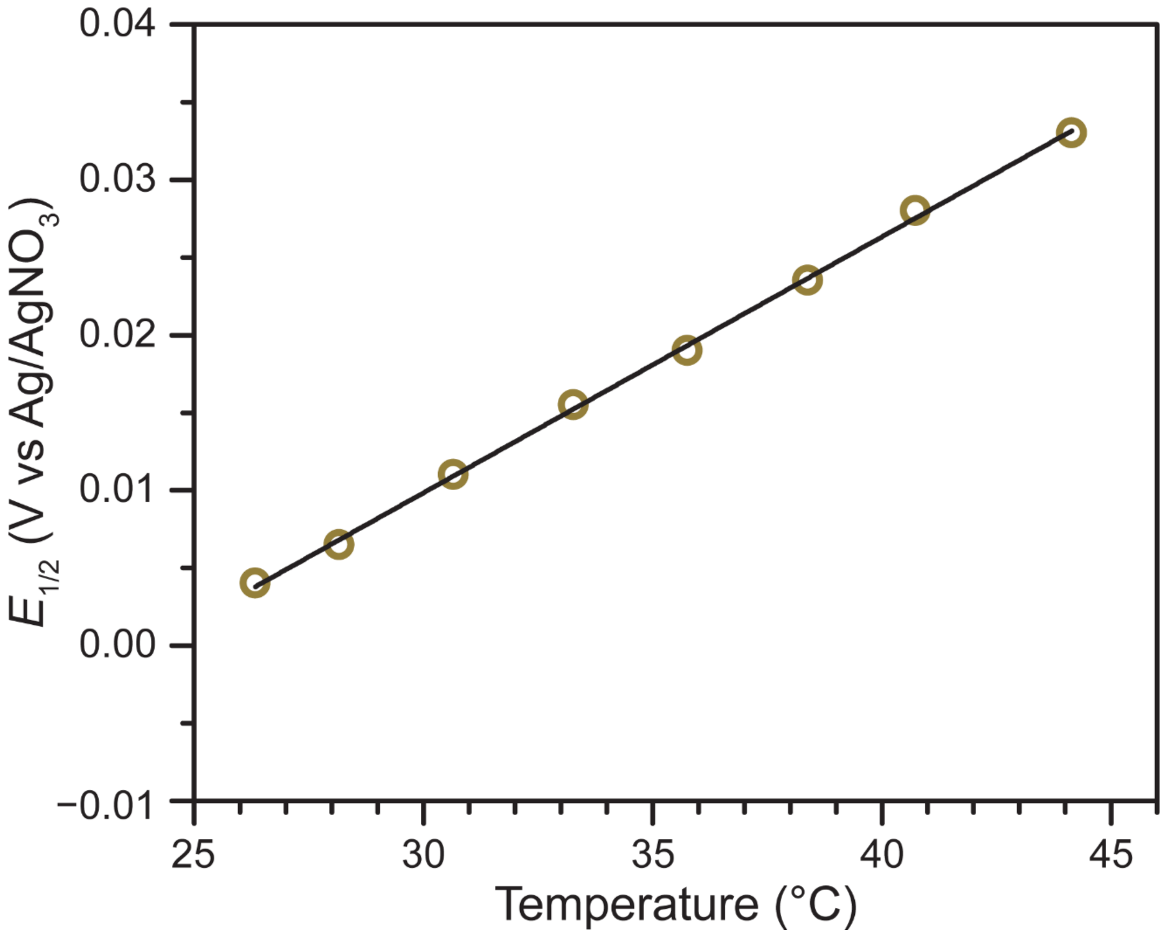

Figure S6. Representative example of a plot of $E_{1/2}$ vs temperature obtained from VT-CV data displayed in Figure S2. The data were collected under isothermal conditions for 2.5 mM of $[Co(bipy)_3]^{2+}$ in MeCN containing 0.1 M (^{n}Bu$_4$N)(PF$_6$) supporting electrolyte using 100 mV s^{-1} scan rate. Gold circles denote experimental data, and the black line corresponds to a linear fit to the data. The slope of the linear fit to the data represents the temperature coefficient for the $[Co(bipy)_3]^{3+}/[Co(bipy)_3]^{2+}$ redox couple as compared to the Ag/AgNO$_3$ reference electrode potential in the given solution conditions.

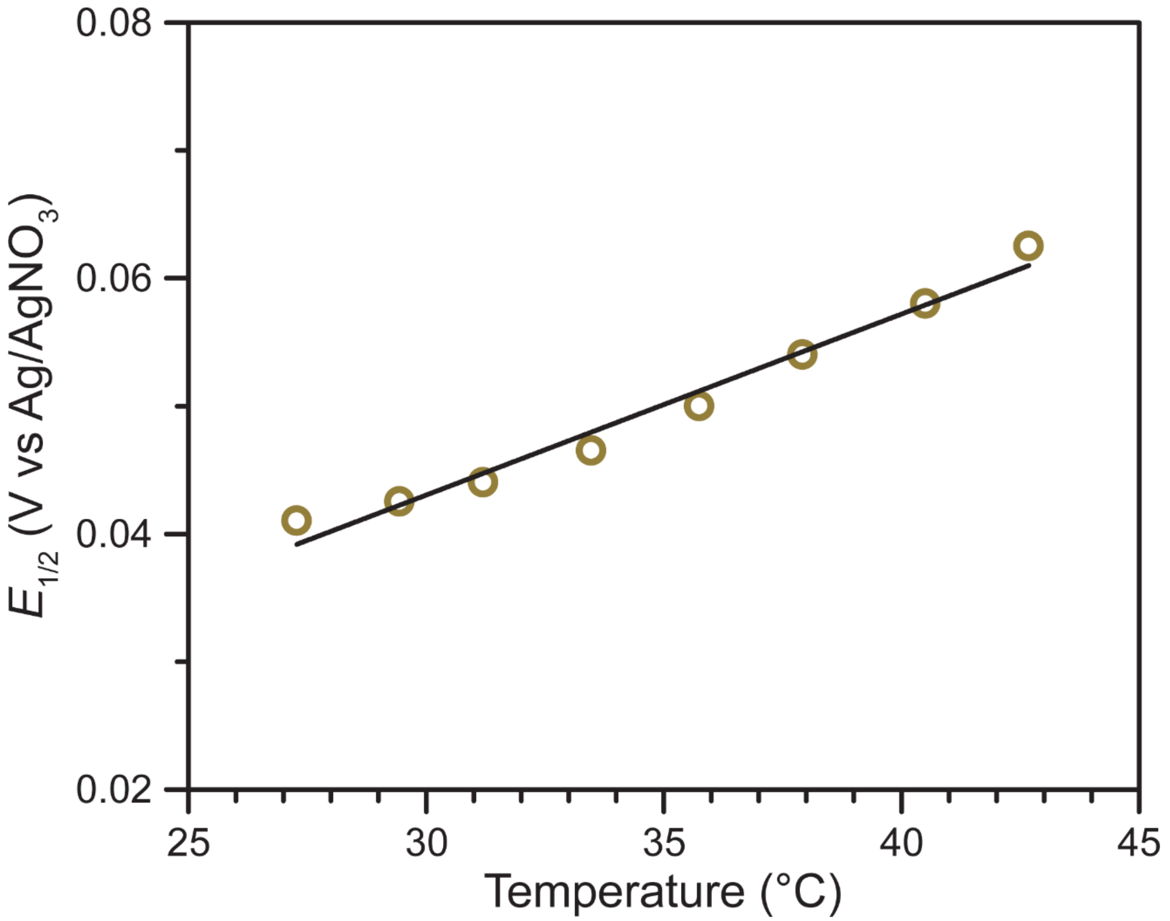

Figure S7. Representative example of a plot of $E_{1/2}$ vs temperature obtained from VT-CV data displayed in Figure S3. The data were collected under isothermal conditions for 2.5 mM of $[Co(bipy)_3]^{3+}$ in MeCN containing 0.1 M KPF_6 supporting electrolyte using 100 mV s^{-1} scan rate. Gold circles denote experimental data, and the black line corresponds to a linear fit to the data. The slope of the linear fit to the data represents the temperature coefficient for the $[Co(bipy)_3]^{3+}/[Co(bipy)_3]^{2+}$ redox couple as compared to the $Ag/AgNO_3$ reference electrode potential in the given solution conditions.

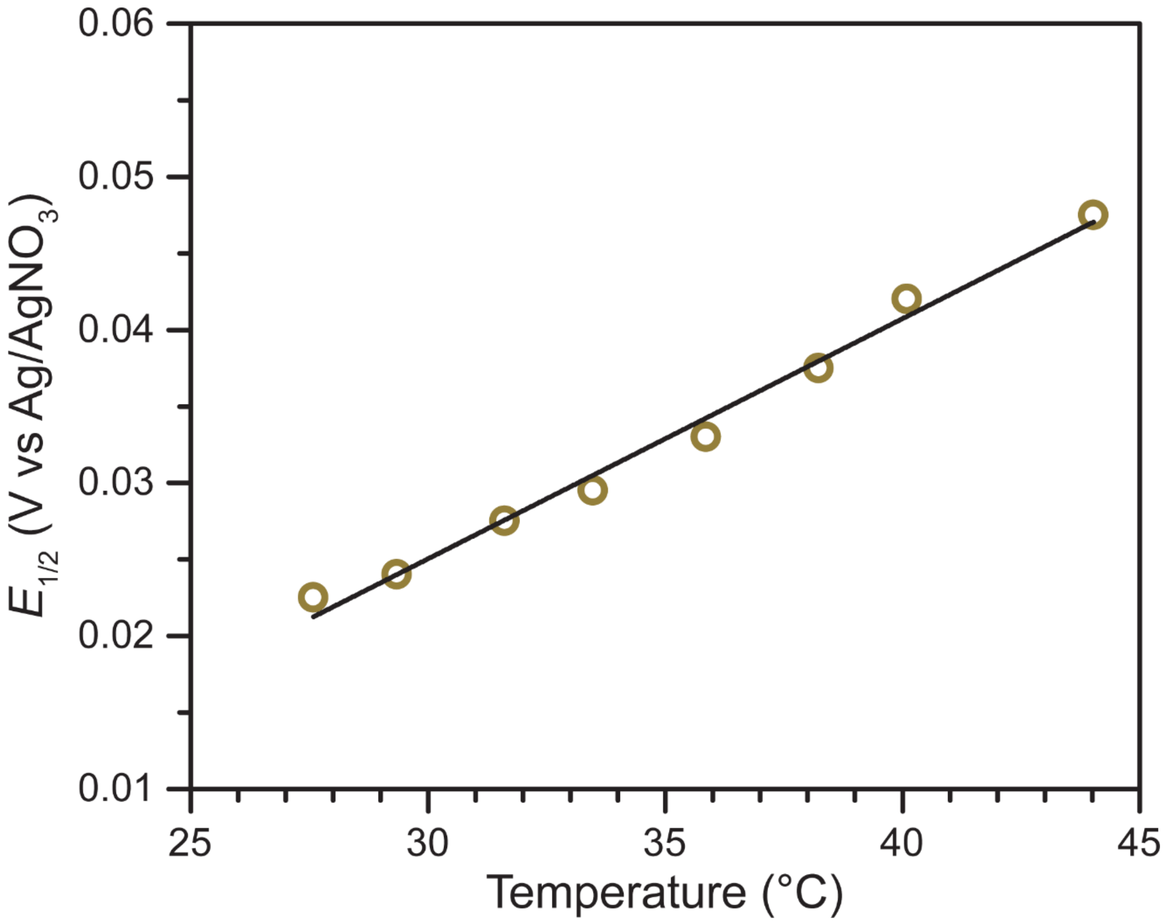

Figure S8. Representative example of a plot of $E_{1/2}$ vs temperature obtained from VT-CV data displayed in Figure S4. The data were collected under isothermal conditions for 2.5 mM of $[Co(bipy)_3]^{3+}$ in MeCN containing 0.1 M ($^{n}Bu_4N$)(PF_6) supporting electrolyte using 100 mV s^{-1} scan rate. Gold circles denote experimental data, and the black line corresponds to a linear fit to the data. The slope of the linear fit to the data represents the temperature coefficient for the $[Co(bipy)_3]^{3+}$/$[Co(bipy)_3]^{2+}$ redox couple as compared to the Ag/$AgNO_3$ reference electrode potential in the given solution conditions.

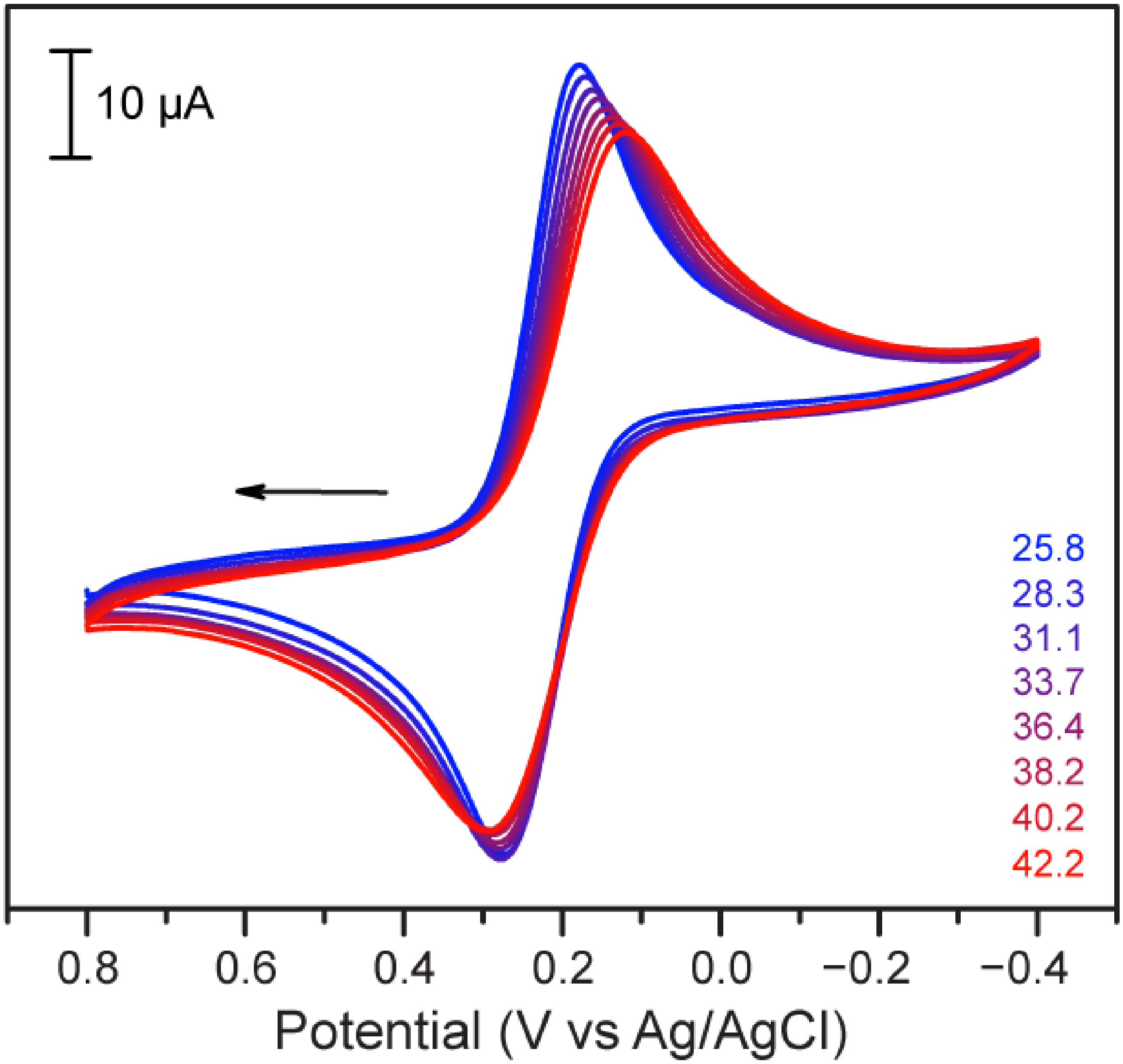

Figure S9. Representative example of VT-CV data collected under isothermal conditions for 2.5 mM of $[Fe(CN)_6]^{4-}$ in water containing 0.1 M KNO_3 supporting electrolyte using 100 mV s^{-1} scan rate. The black arrow denotes the scan direction, and the colored numbers denote the solution temperatures for respective voltammograms in °C. Pt, glass Ag/AgCl (filled with a 3 M aqueous KCl solution), and Pt mesh were used as working, reference, and counter electrodes, respectively.

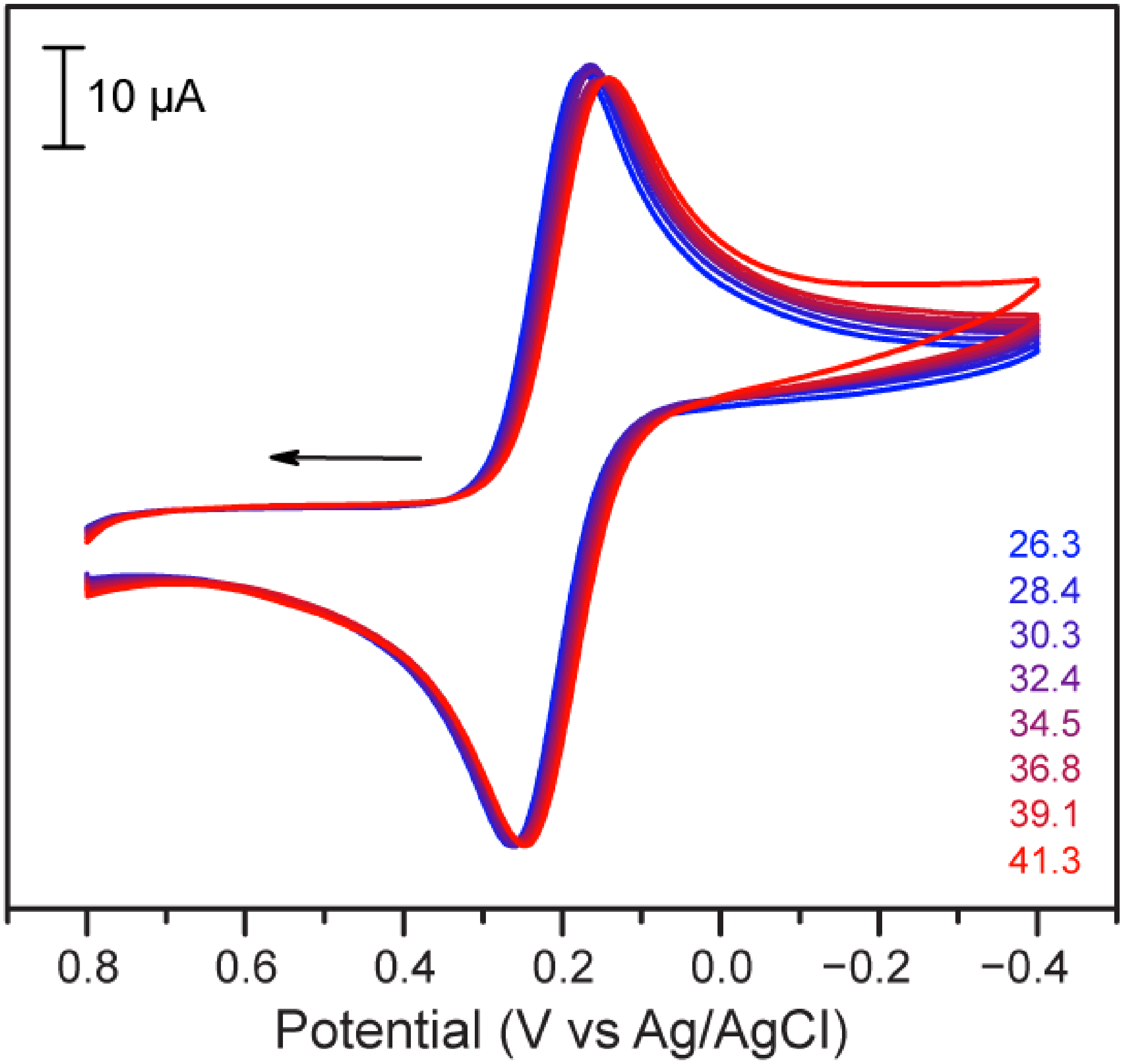

Figure S10. Representative example of VT-CV data collected under isothermal conditions for 2.5 mM of $[Fe(CN)_6]^{3-}$ in water containing 0.1 M KNO_3 supporting electrolyte using 100 mV s^{-1} scan rate. The black arrow denotes the scan direction, and the colored numbers denote the solution temperatures for respective voltammograms in °C. Pt, glass Ag/AgCl (filled with a 3 M aqueous KCl solution), and Pt mesh were used as working, reference, and counter electrodes, respectively.

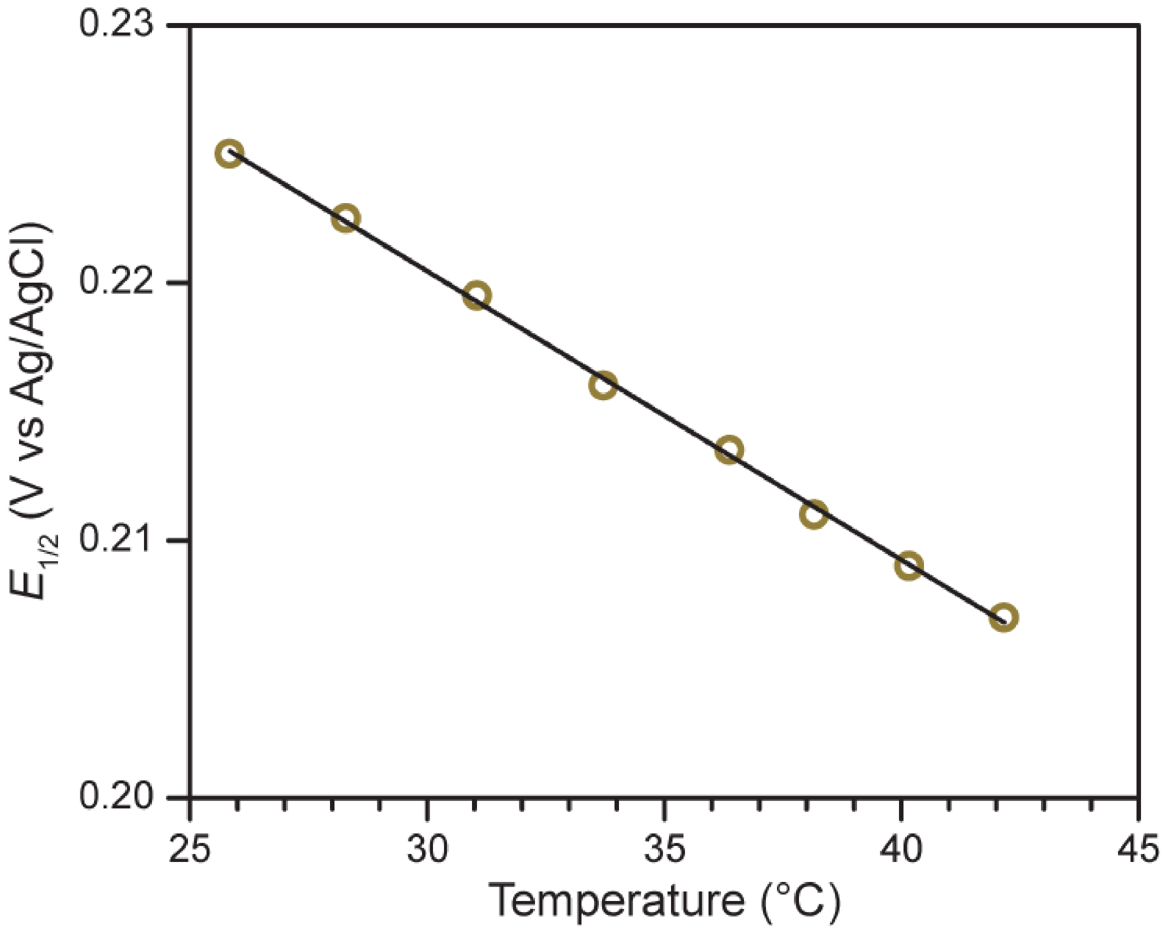

Figure S11. Representative example of a plot of $E_{1/2}$ vs temperature obtained from VT-CV data displayed in Figure S9. The data were collected under isothermal conditions for 2.5 mM of $[Fe(CN)_6]^{4-}$ in water containing 0.1 M KNO_3 supporting electrolyte using 100 mV s^{-1} scan rate. Gold circles denote experimental data, and the black line corresponds to a linear fit to the data. The slope of the linear fit to the data represents the temperature coefficient for the $[Fe(CN)_6]^{3-}/[Fe(CN)_6]^{4-}$ redox couple as compared to the glass Ag/AgCl (filled with a 3 M aqueous KCl solution) reference electrode potential in the given solution conditions.

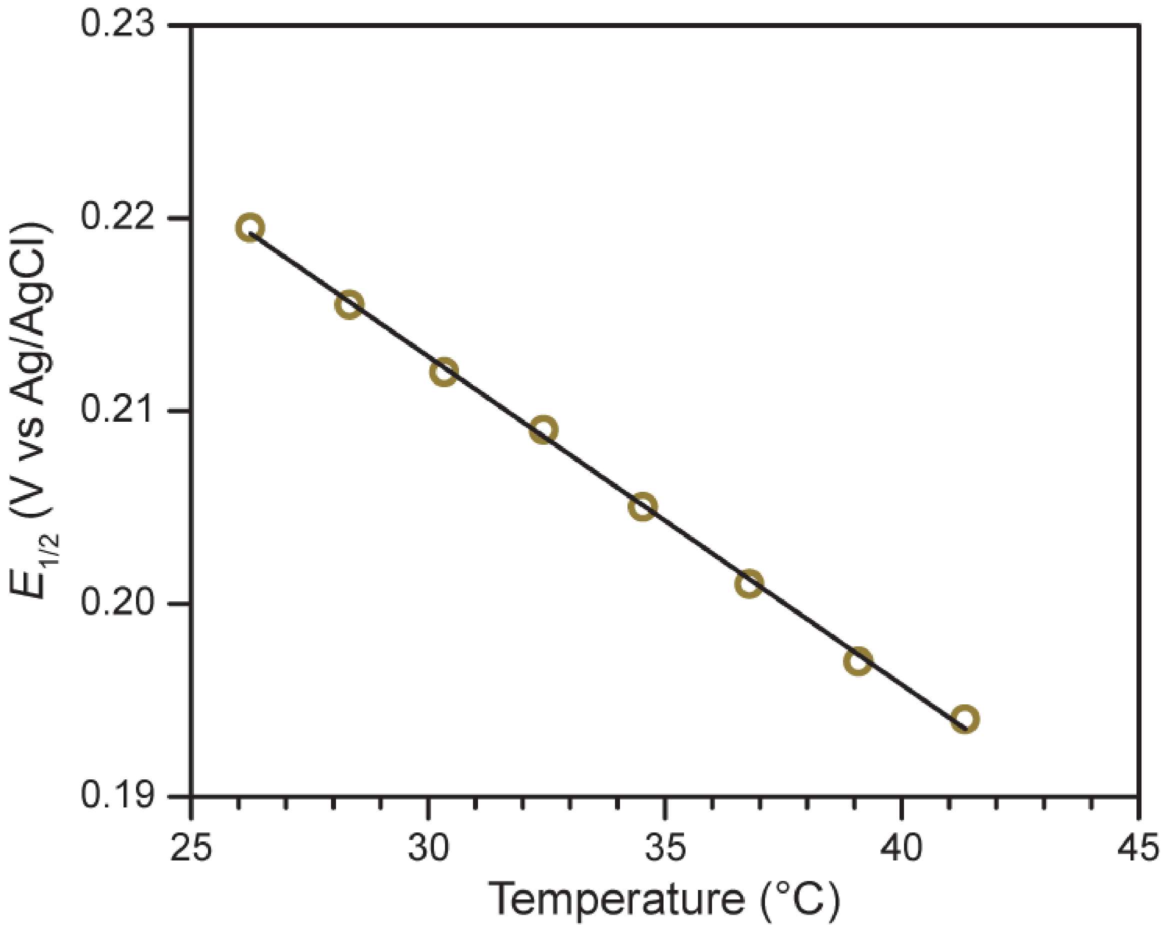

Figure S12. Representative example of a plot of $E_{1/2}$ vs temperature obtained from VT-CV data displayed in Figure S10. The data were collected under isothermal conditions for 2.5 mM of $[Fe(CN)_6]^{3-}$ in water containing 0.1 M KNO_3 supporting electrolyte using 100 mV s^{-1} scan rate. Gold circles denote experimental data, and the black line corresponds to a linear fit to the data. The slope of the linear fit to the data represents the temperature coefficient for the $[Fe(CN)_6]^{3-}/[Fe(CN)_6]^{4-}$ redox couple as compared to the glass Ag/AgCl (filled with a 3 M aqueous KCl solution) reference electrode potential in the given solution conditions.

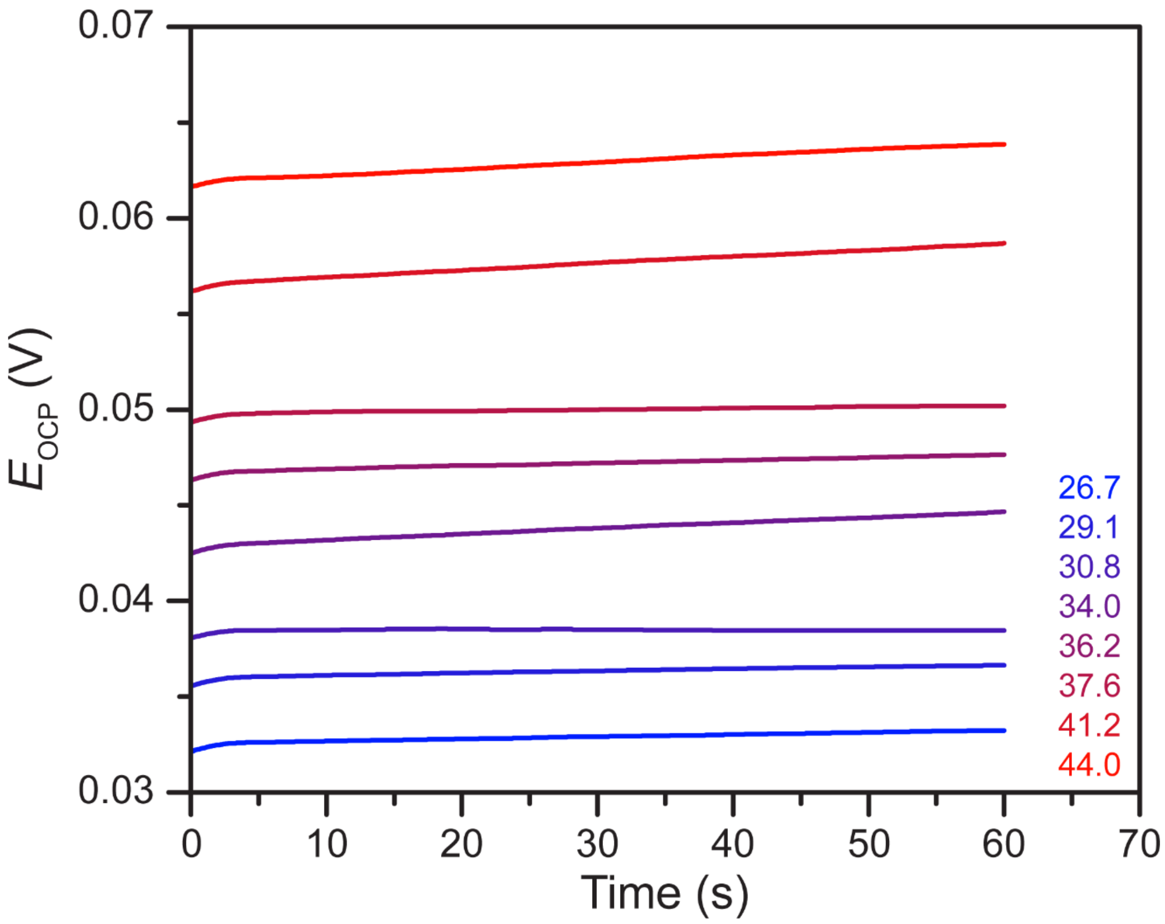

Figure S13. Representative example of VT-OCP data collected under isothermal conditions for an equimolar solution of $[Co(bipy)_3]^{2+}$ and $[Co(bipy)_3]^{3+}$ (2.5 mM each) in MeCN containing 0.1 M KPF_6 supporting electrolyte. The colored numbers denote the solution temperatures for respective E_{OCP} traces in °C. Glassy carbon, $Ag/AgNO_3$, and Pt mesh were used as working, reference, and counter electrodes, respectively.

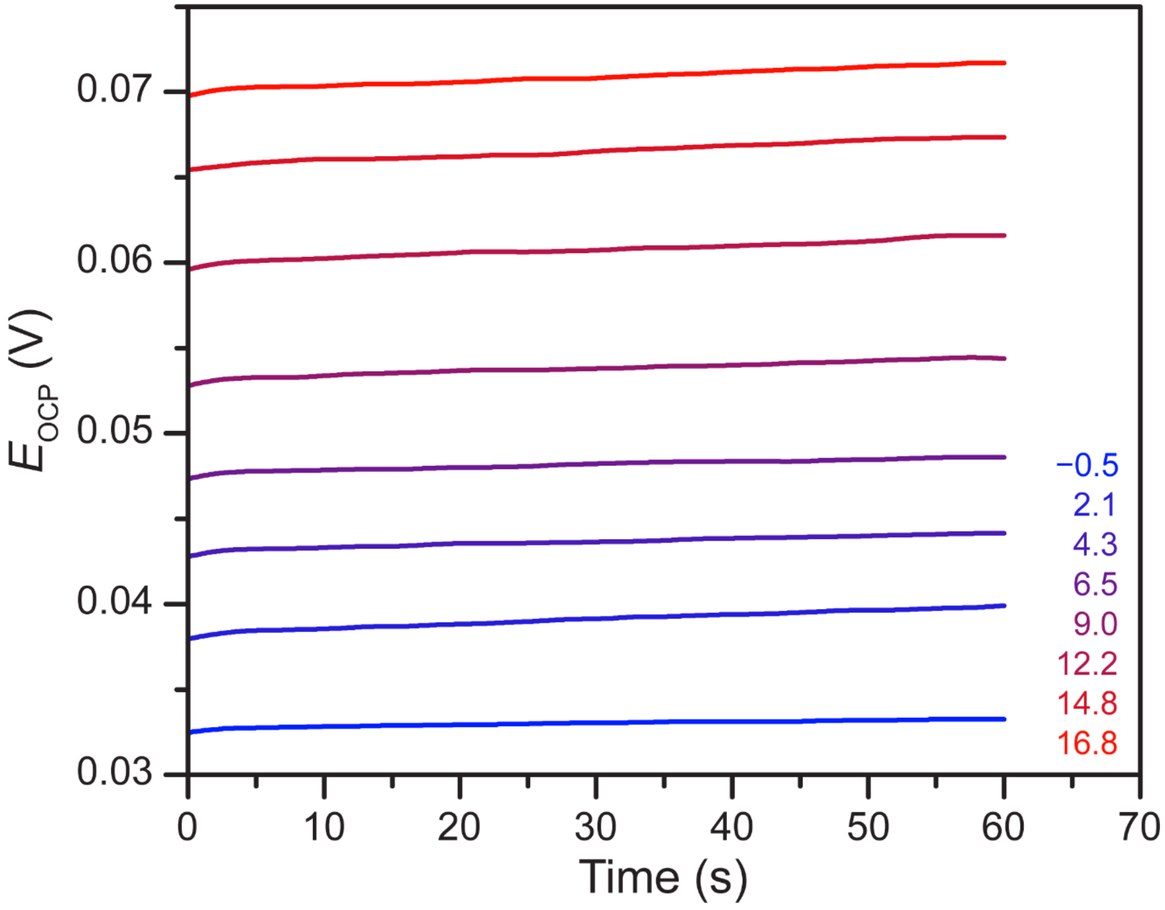

Figure S14. Representative example of VT-OCP data collected under nonisothermal conditions for an equimolar solution of $[Co(bipy)_3]^{2+}$ and $[Co(bipy)_3]^{3+}$ (2.5 mM each) in MeCN containing 0.1 M KPF_6 supporting electrolyte. The colored numbers denote the temperature difference between the heated and nonheated solutions for respective E_{OCP} traces in °C. Glassy carbon, Ag/$AgNO_3$, and Pt mesh were used as working, reference, and counter electrodes, respectively.

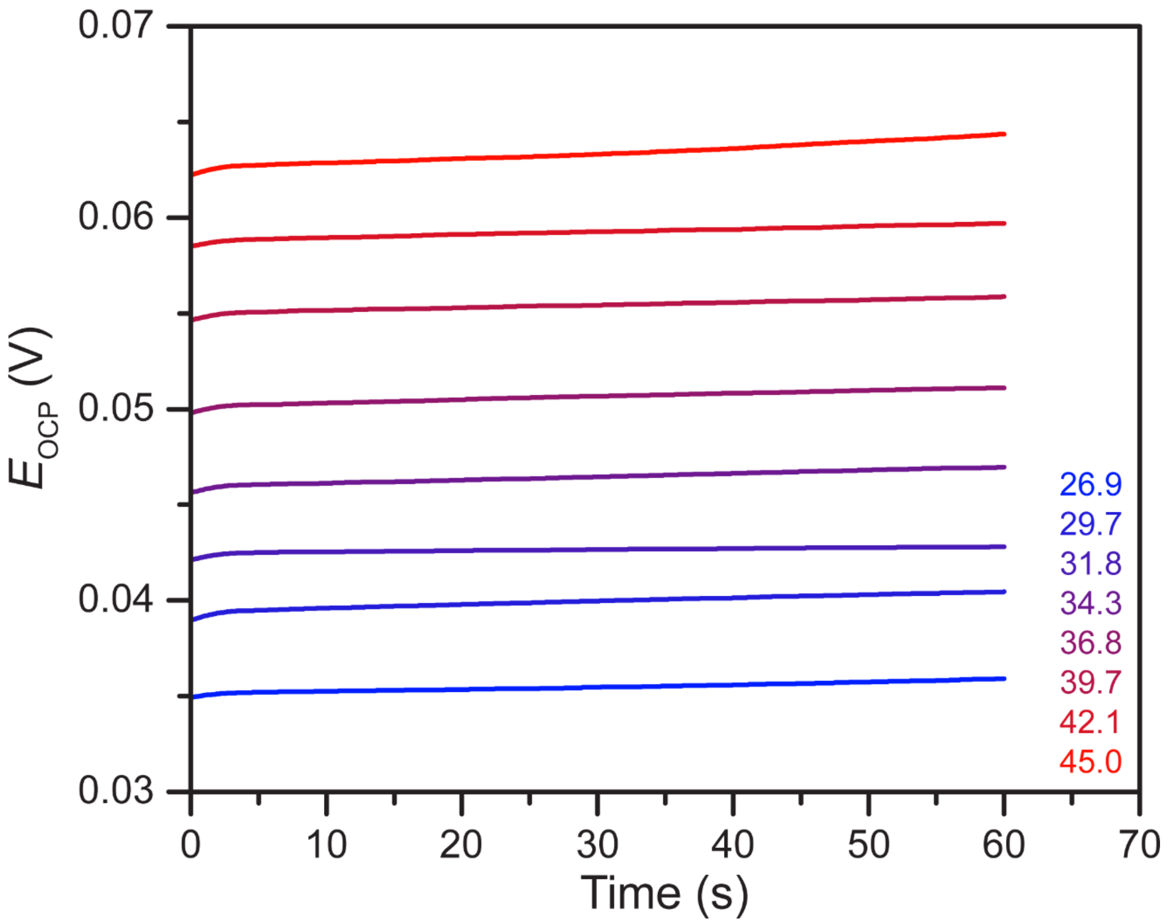

Figure S15. Representative example of VT-OCP data collected under isothermal conditions for an equimolar solution of $[Co(bipy)_3]^{2+}$ and $[Co(bipy)_3]^{3+}$ (2.5 mM each) in MeCN containing 0.1 M $(^{n}Bu_4N)(PF_6)$ supporting electrolyte. The colored numbers denote the solution temperatures for respective E_{OCP} traces in °C. Glassy carbon, $Ag/AgNO_3$, and Pt mesh were used as working, reference, and counter electrodes, respectively.

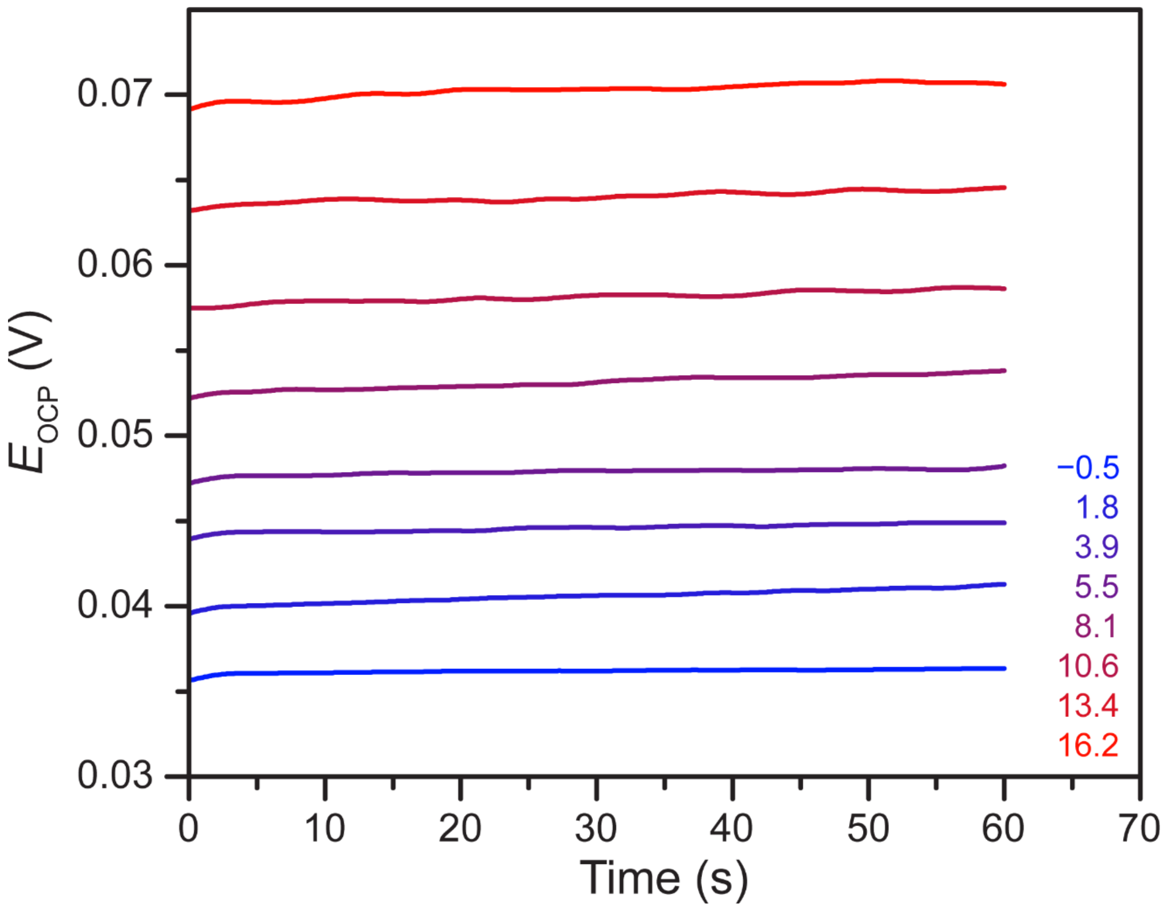

Figure S16. Representative example of VT-OCP data collected under nonisothermal conditions for an equimolar solution of $[Co(bipy)_3]^{2+}$ and $[Co(bipy)_3]^{3+}$ (2.5 mM each) in MeCN containing 0.1 M (^{n}Bu$_4$N)(PF$_6$) supporting electrolyte. The colored numbers denote the temperature difference between the heated and nonheated solutions for respective E_{OCP} traces in °C. Glassy carbon, Ag/AgNO$_3$, and Pt mesh were used as working, reference, and counter electrodes, respectively.

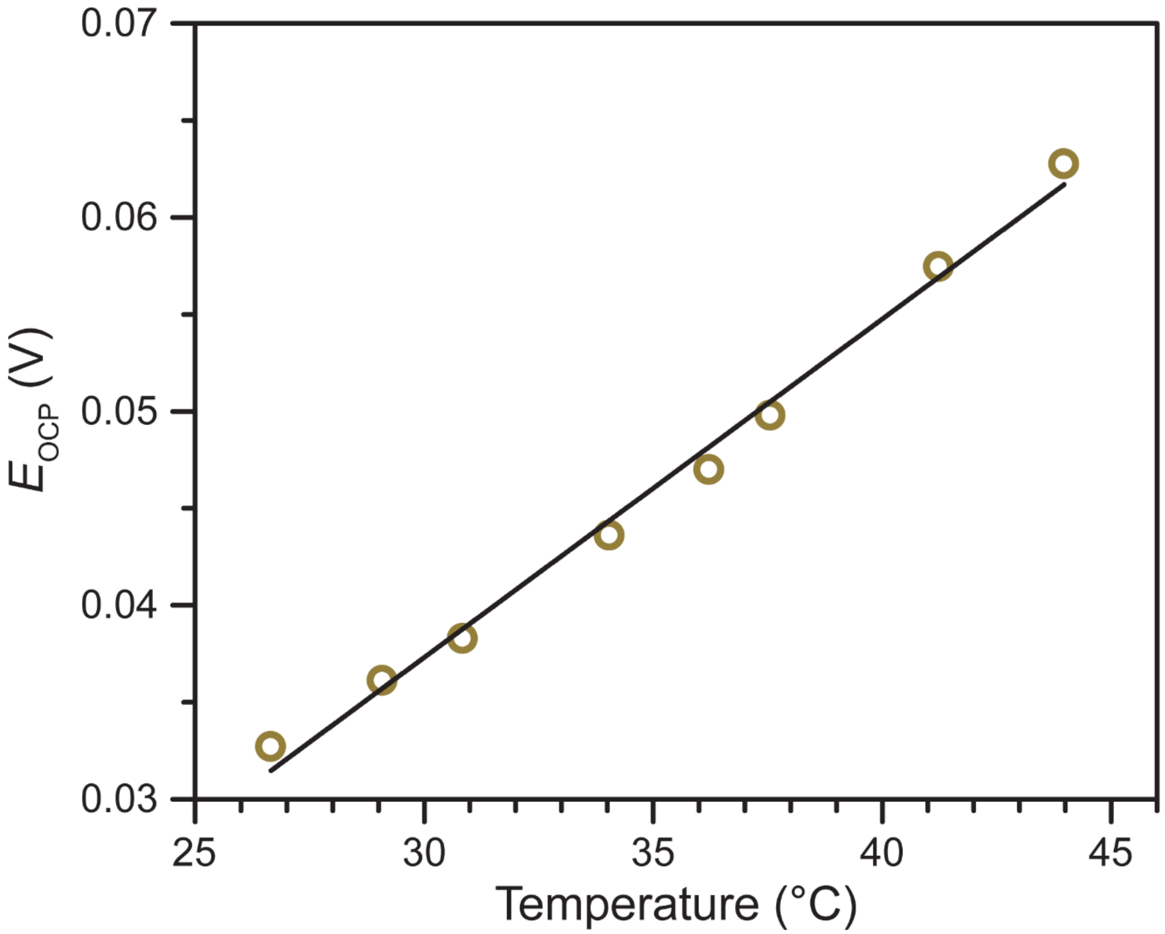

Figure S17. Representative example of a plot of E_{OCP} vs temperature obtained from isothermal VT-OCP data displayed in Figure S13. The data were collected for an equimolar solution of $[Co(bipy)_3]^{2+}$ and $[Co(bipy)_3]^{3+}$ (2.5 mM each) in MeCN containing 0.1 M KPF_6 supporting electrolyte. Gold circles denote experimental data, and the black line corresponds to a linear fit to the data. The slope of the linear fit to the data represents the temperature coefficient for the $[Co(bipy)_3]^{3+}/[Co(bipy)_3]^{2+}$ redox couple as compared to the $Ag/AgNO_3$ reference electrode potential in the given solution conditions.

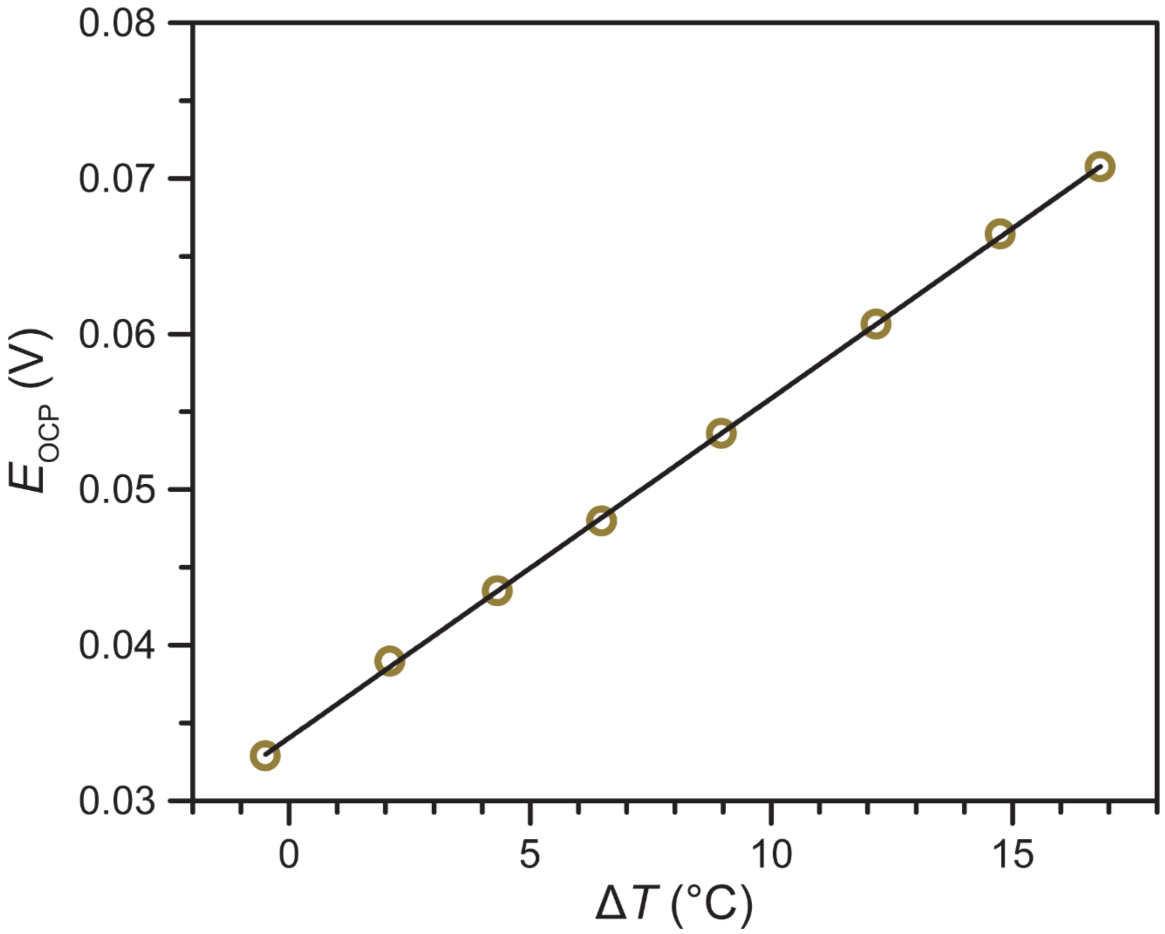

Figure S18. Representative example of a plot of E_{OCP} vs ΔT obtained from nonisothermal VT-OCP data displayed in Figure S14. The data were collected for an equimolar solution of $[Co(bipy)_3]^{2+}$ and $[Co(bipy)_3]^{3+}$ (2.5 mM each) in MeCN containing 0.1 M KPF_6 supporting electrolyte. Gold circles denote experimental data, and the black line corresponds to a linear fit to the data. The slope of the linear fit to the data represents the true temperature coefficient for the $[Co(bipy)_3]^{3+}/[Co(bipy)_3]^{2+}$ redox couple in the given solution conditions.

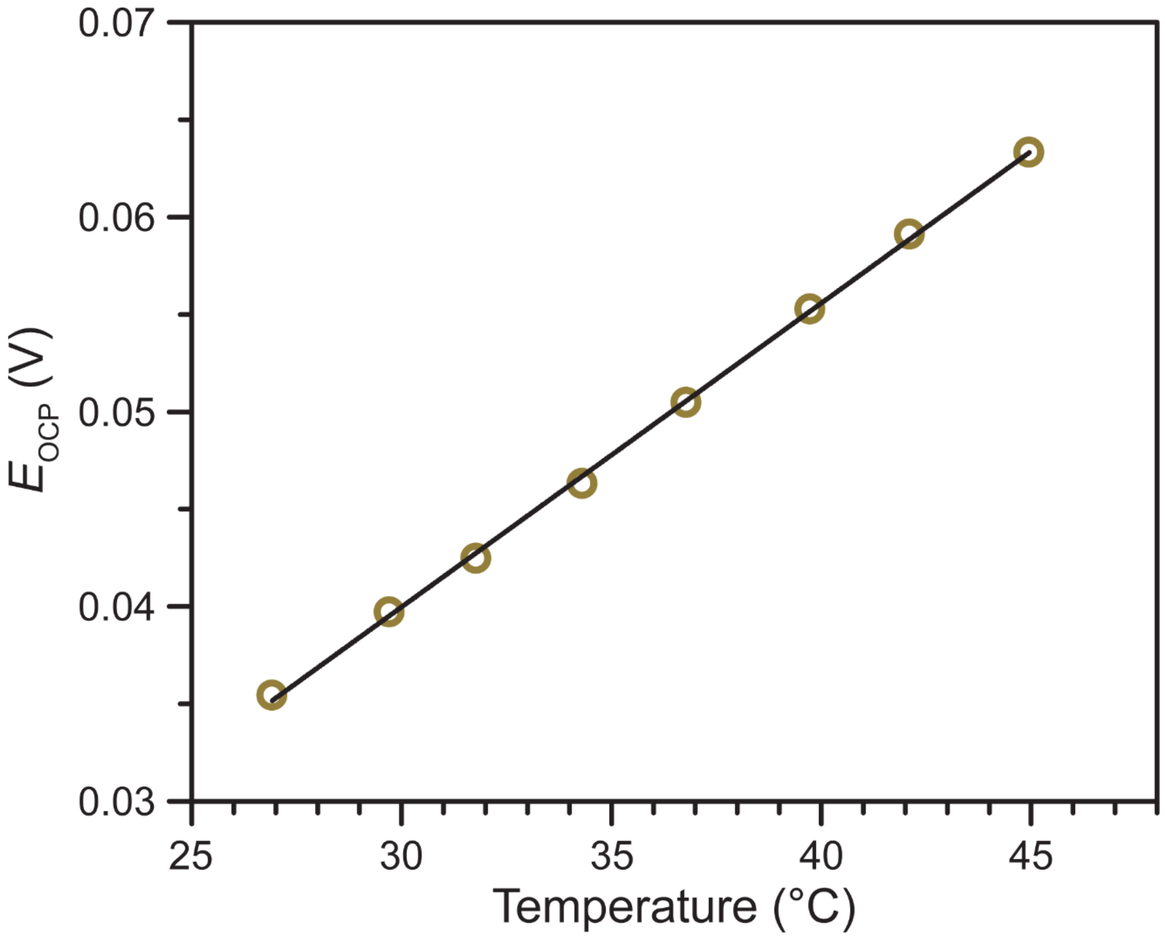

Figure S19. Representative example of a plot of E_{OCP} vs temperature obtained from isothermal VT-OCP data displayed in Figure S15. The data were collected for an equimolar solution of $[Co(bipy)_3]^{2+}$ and $[Co(bipy)_3]^{3+}$ (2.5 mM each) in MeCN containing 0.1 M (^{n}Bu$_4$N)(PF$_6$) supporting electrolyte. Gold circles denote experimental data, and the black line corresponds to a linear fit to the data. The slope of the linear fit to the data represents the temperature coefficient for the $[Co(bipy)_3]^{3+}/[Co(bipy)_3]^{2+}$ redox couple as compared to the Ag/AgNO$_3$ reference electrode potential in the given solution conditions.

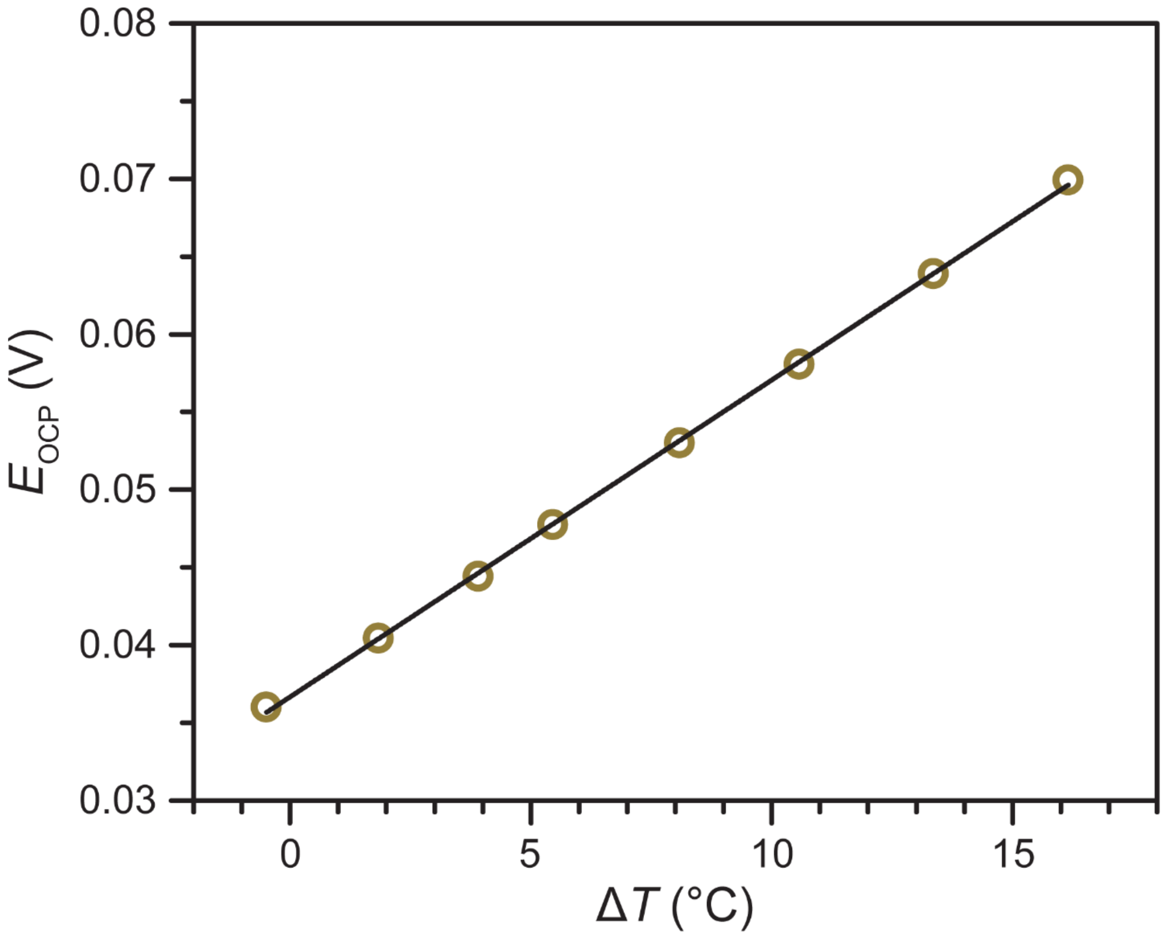

Figure S20. Representative example of a plot of E_{OCP} vs ΔT obtained from nonisothermal VT-OCP data displayed in Figure S16. The data were collected for an equimolar solution of $[Co(bipy)_3]^{2+}$ and $[Co(bipy)_3]^{3+}$ (2.5 mM each) in MeCN containing 0.1 M $(^nBu_4N)(PF_6)$ supporting electrolyte. Gold circles denote experimental data, and the black line corresponds to a linear fit to the data. The slope of the linear fit to the data represents the true temperature coefficient for the $[Co(bipy)_3]^{3+}/[Co(bipy)_3]^{2+}$ redox couple in the given solution conditions.

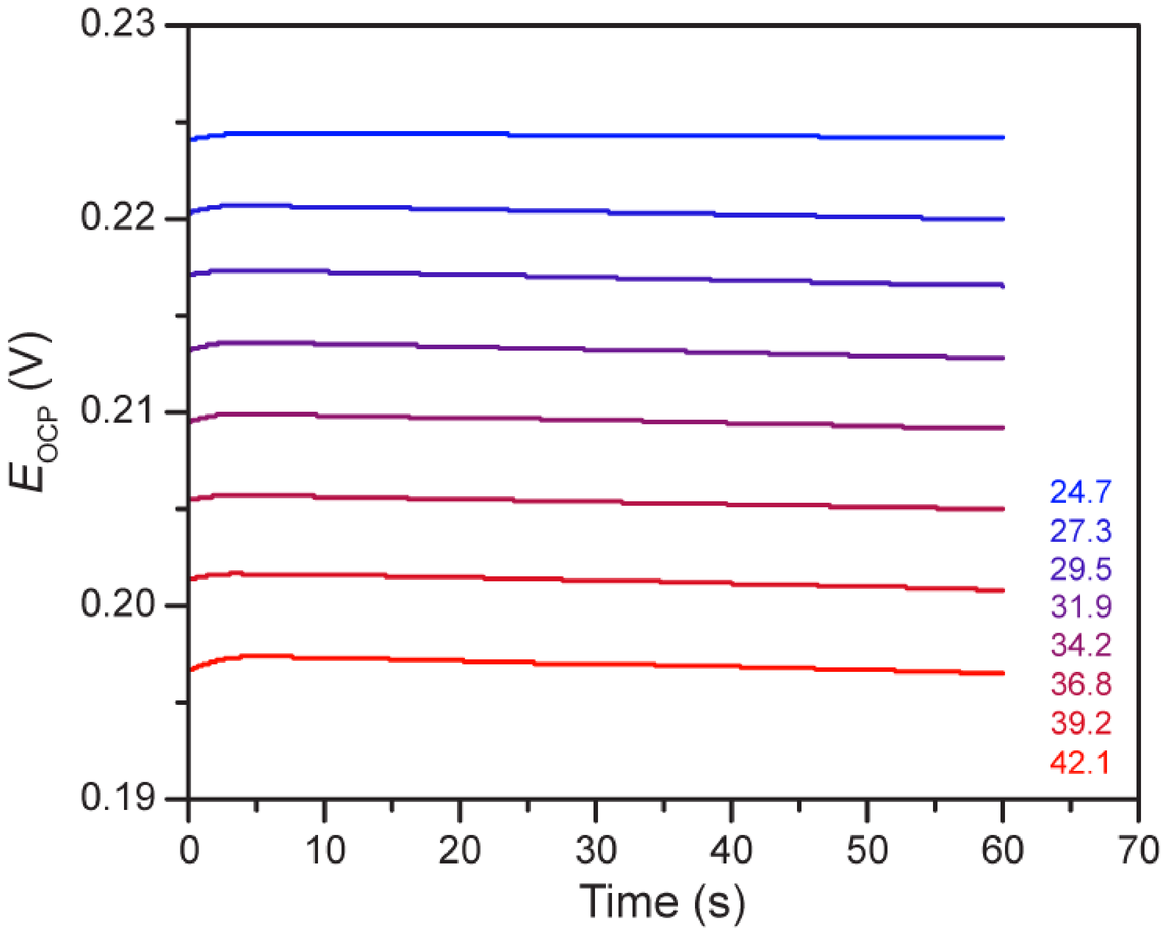

Figure S21. Representative example of VT-OCP data collected under isothermal conditions for an equimolar solution of $[Fe(CN)_6]^{4-}$ and $[Fe(CN)_6]^{3-}$ (2.5 mM each) in water containing 0.1 M KNO_3 supporting electrolyte. The colored numbers denote the solution temperatures for respective E_{OCP} traces in °C. Pt, glass Ag/AgCl (filled with a 3 M aqueous KCl solution), and Pt mesh were used as working, reference, and counter electrodes, respectively.

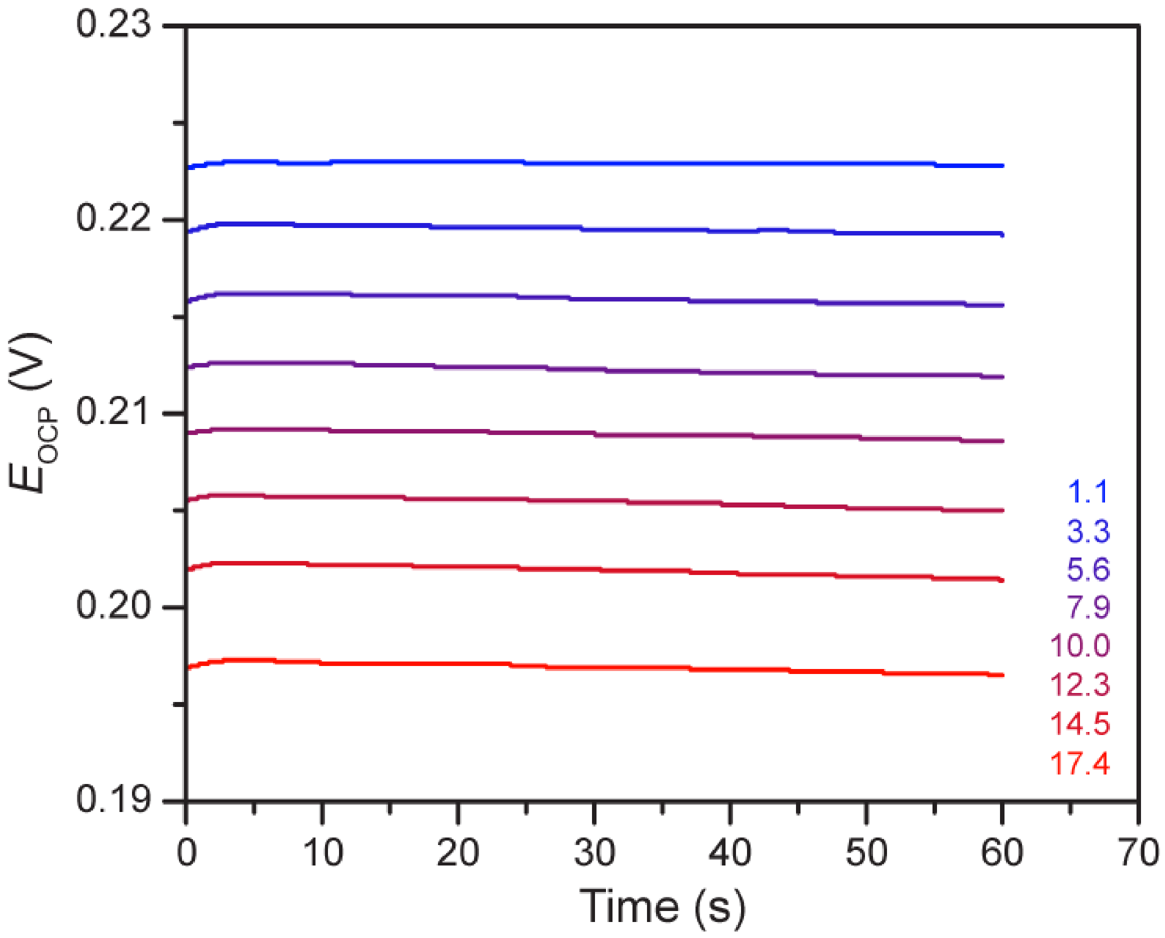

Figure S22. Representative example of VT-OCP data collected under nonisothermal conditions for an equimolar solution of $[Fe(CN)_6]^{4-}$ and $[Fe(CN)_6]^{3-}$ (2.5 mM each) in water containing 0.1 M KNO_3 supporting electrolyte. The colored numbers denote the temperature difference between the heated and nonheated solutions for respective E_{OCP} traces in °C. Pt, glass Ag/AgCl (filled with a 3 M aqueous KCl solution), and Pt mesh were used as working, reference, and counter electrodes, respectively.

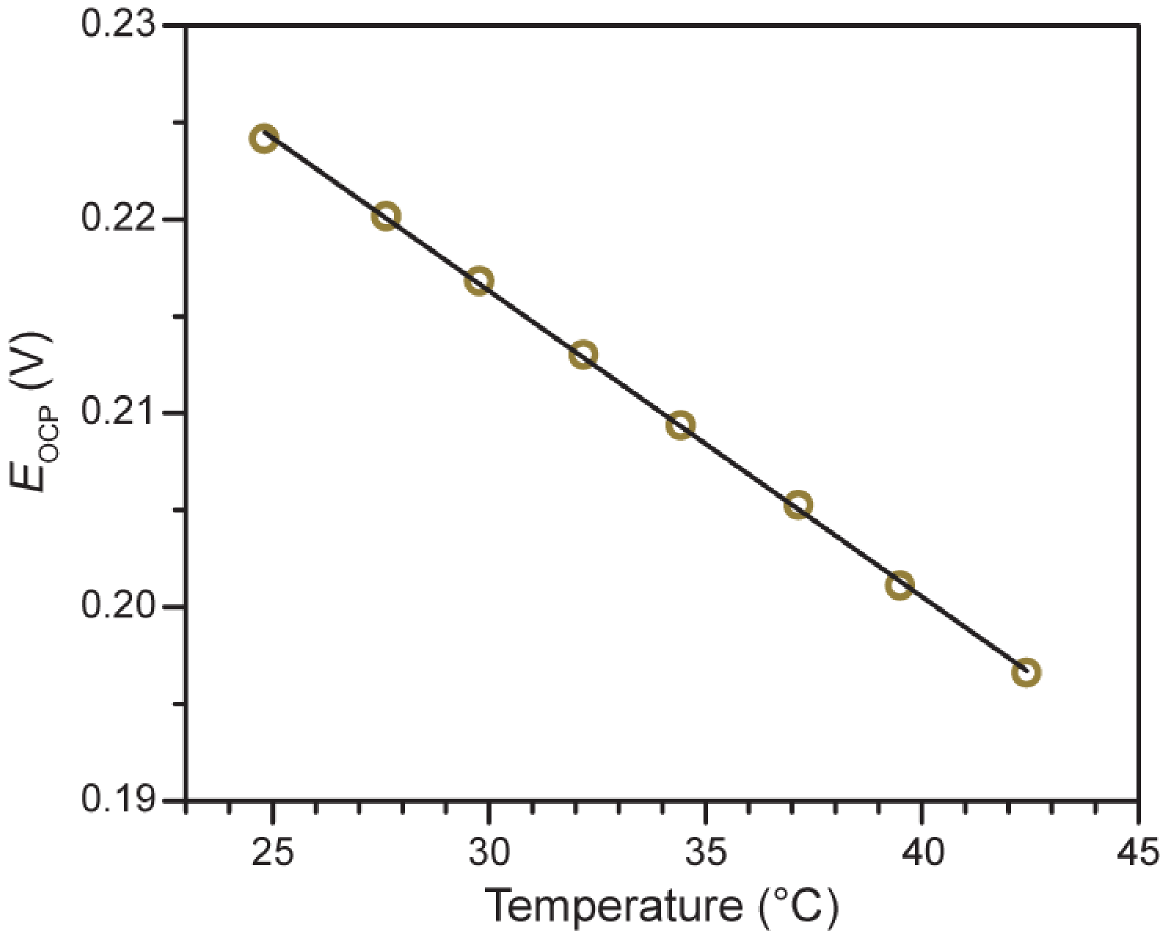

Figure S23. Representative example of a plot of E_{OCP} vs temperature obtained from isothermal VT-OCP data displayed in Figure S21. The data were collected for an equimolar solution of $[Fe(CN)_6]^{4-}$ and $[Fe(CN)_6]^{3-}$ (2.5 mM each) in water containing 0.1 M KNO_3 supporting electrolyte. Gold circles denote experimental data, and the black line corresponds to a linear fit to the data. The slope of the linear fit to the data represents the temperature coefficient for the $[Fe(CN)_6]^{3-}$/$[Fe(CN)_6]^{4-}$ redox couple as compared to the glass Ag/AgCl (filled with a 3 M aqueous KCl solution) reference electrode potential in the given solution conditions.

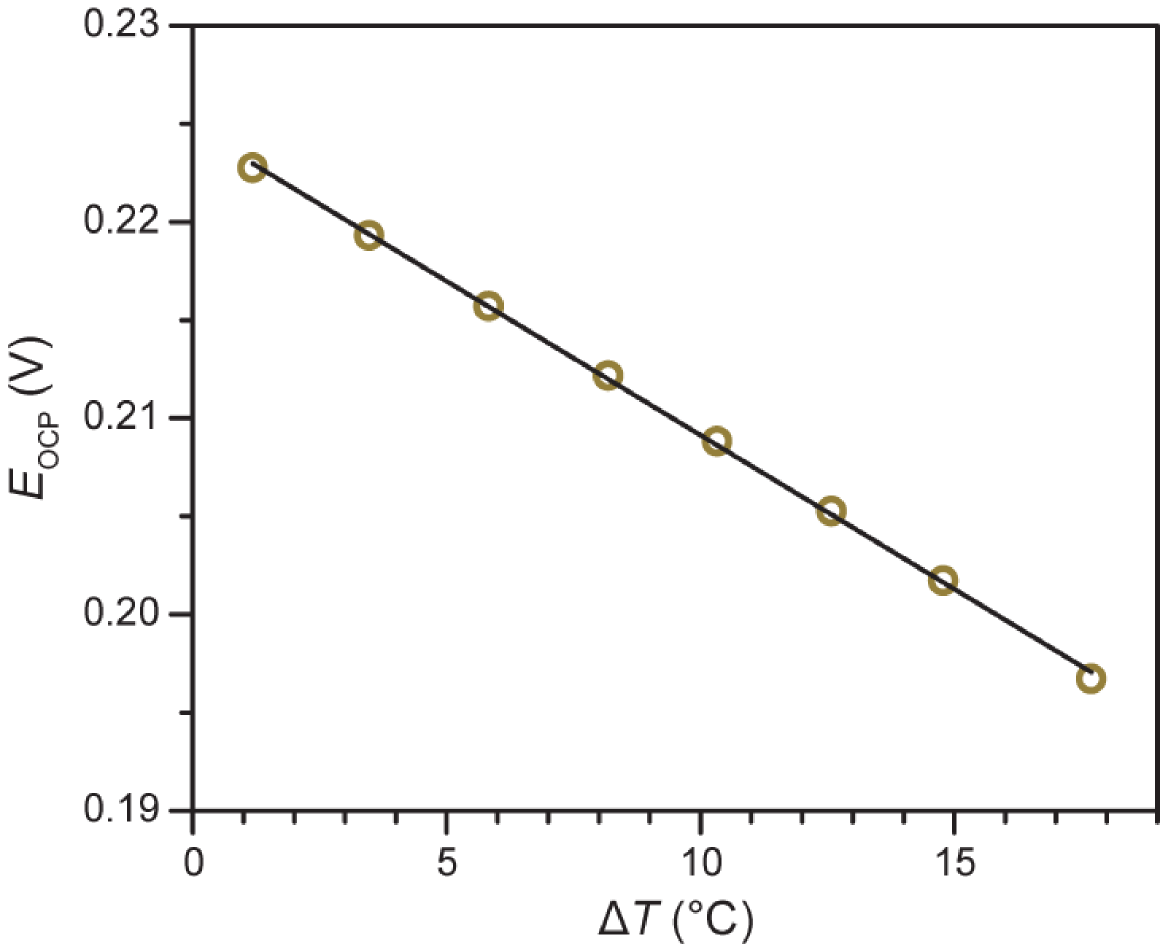

Figure S24. Representative example of a plot of E_{OCP} vs Δ*T* obtained from nonisothermal VT-OCP data displayed in Figure S22. The data were collected for an equimolar solution of $[Fe(CN)_6]^{4-}$ and $[Fe(CN)_6]^{3-}$ (2.5 mM each) in water containing 0.1 M KNO_3 supporting electrolyte. Gold circles denote experimental data, and the black line corresponds to a linear fit to the data. The slope of the linear fit to the data represents the true temperature coefficient for the $[Fe(CN)_6]^{3-}/[Fe(CN)_6]^{4-}$ redox couple in the given solution conditions.

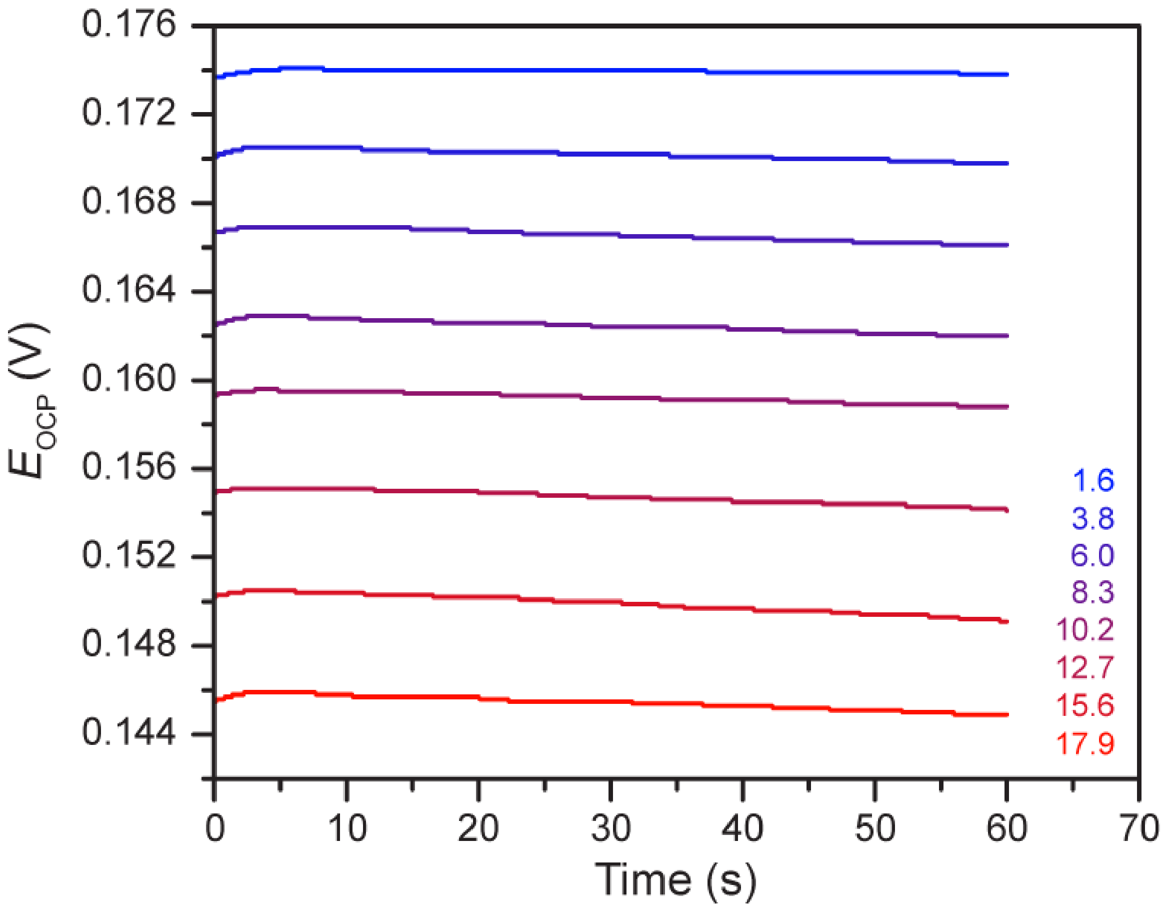

Figure S25. Representative example of VT-OCP data collected under nonisothermal conditions for an equimolar solution of $[Fe(CN)_6]^{4-}$ and $[Fe(CN)_6]^{3-}$ (2.5 mM each) in water containing 0.1 M KNO_3 supporting electrolyte. The colored numbers denote the temperature difference between the heated and nonheated solutions for respective E_{OCP} traces in °C. Pt, leak-free Ag/AgCl, and Pt mesh were used as working, reference, and counter electrodes, respectively.

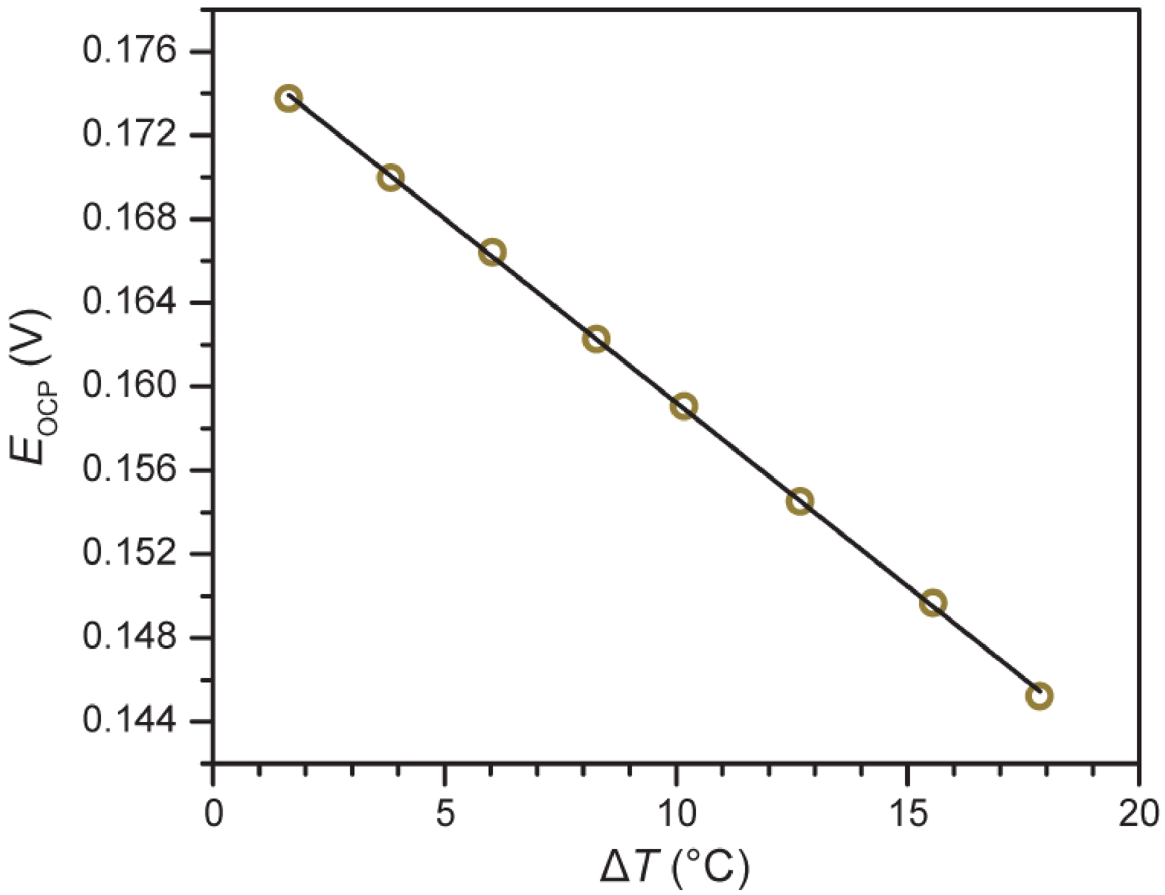

Figure S26. Representative example of a plot of E_{OCP} vs ΔT obtained from nonisothermal VT-OCP data displayed in Figure S25. The data were collected for an equimolar solution of $[Fe(CN)_6]^{4-}$ and $[Fe(CN)_6]^{3-}$ (2.5 mM each) in water containing 0.1 M KNO_3 supporting electrolyte. Gold circles denote experimental data, and the black line corresponds to a linear fit to the data. The slope of the linear fit to the data represents the true temperature coefficient for the $[Fe(CN)_6]^{3-}/[Fe(CN)_6]^{4-}$ redox couple in the given solution conditions.

D. Supplementary Tables

Table S1. Summary of variable-temperature electrochemical data obtained for the $[Co(bipy)_3]^{3+}/[Co(bipy)_3]^{2+}$ redox couple in MeCN containing 0.1 M KPF_6 supporting electrolyte.[a]

**Analyte**	**Method**	**Temperature Conditions**[b]	**α**[c] **(mV K^{-1})**	**ΔS_{redox}**[d] **(J K^{-1} mol^{-1})**
$[Co(bipy)_3]^{2+}$	VT-CV	Isothermal	2.1(1)	203(10)
$[Co(bipy)_3]^{3+}$	VT-CV	Isothermal	2.0(2)	193(20)
$[Co(bipy)_3]^{2+}$ + $[Co(bipy)_3]^{3+}$	VT-OCP	Isothermal	2.2(2)	212(20)
$[Co(bipy)_3]^{2+}$ + $[Co(bipy)_3]^{3+}$	VT-OCP	Nonisothermal	2.2(1)	212(10)

[a]All data were collected using three-electrode setup with glassy carbon, $Ag/AgNO_3$, and Pt mesh as working, reference, and counter electrodes, respectively. [b]Isothermal and nonisothermal measurements were carried out using single-compartment and three-compartment electrochemical glass cells, respectively, as described in the Experimental Section. [c]Temperature coefficient (α) calculated using equations S1–S3, with the temperature coefficient of the reference electrode potential considered for measurements conducted under isothermal conditions; average values from 4–7 independent measurements and error bars denote standard deviations of those measurements. [d]Change in entropy associated with the given redox reaction, calculated using equation S4; errors were estimated using error propagation of the average temperature coefficient values.

Table S2. Summary of variable-temperature electrochemical data obtained for the $[Co(bipy)_3]^{3+}/[Co(bipy)_3]^{2+}$ redox couple in MeCN containing 0.1 M $(^nBu_4N)(PF_6)$ supporting electrolyte.[a]

Analyte	Method	Temperature Conditions[b]	α^c (mV K^{-1})	$\Delta S_{redox}{}^d$ (J K^{-1} mol^{-1})
$[Co(bipy)_3]^{2+}$	VT-CV	Isothermal	2.1(1)	203(10)
$[Co(bipy)_3]^{3+}$	VT-CV	Isothermal	2.0(1)	193(10)
$[Co(bipy)_3]^{2+}$ + $[Co(bipy)_3]^{3+}$	VT-OCP	Isothermal	2.0(1)	193(10)
$[Co(bipy)_3]^{2+}$ + $[Co(bipy)_3]^{3+}$	VT-OCP	Nonisothermal	2.1(2)	203(20)

[a]All data were collected using three-electrode setup with glassy carbon, $Ag/AgNO_3$, and Pt mesh as working, reference, and counter electrodes, respectively. [b]Isothermal and nonisothermal measurements were carried out using single-compartment and three-compartment electrochemical glass cells, respectively, as described in the Experimental Section. [c]Temperature coefficient (α) calculated using equations S1–S3, with the temperature coefficient of the reference electrode potential considered for measurements conducted under isothermal conditions; average values from 3 independent measurements and error bars denote standard deviations of those measurements. [d]Change in entropy associated with the given redox reaction, calculated using equation S4; errors were estimated using error propagation of the average temperature coefficient values.

Table S3. Summary of variable-temperature electrochemical data obtained for the $[Fe(CN)_6]^{3-}/[Fe(CN)_6]^{4-}$ redox couple in water containing 0.1 M KNO_3 supporting electrolyte.[a]

Analyte	Method	Temperature Conditions[b]	α^c (mV K^{-1})	$\Delta S_{redox}{}^d$ (J K^{-1} mol^{-1})
$[Fe(CN)_6]^{4-e}$	VT-CV	Isothermal	−1.0(1)	−96(10)
$[Fe(CN)_6]^{3-e}$	VT-CV	Isothermal	−1.6(1)	−154(10)
$[Fe(CN)_6]^{4-}$ + $[Fe(CN)_6]^{3-e}$	VT-OCP	Isothermal	−1.4(1)	−135(10)
$[Fe(CN)_6]^{4-}$ + $[Fe(CN)_6]^{3-e}$	VT-OCP	Nonisothermal	−1.4(2)	−135(20)
$[Fe(CN)_6]^{4-}$ + $[Fe(CN)_6]^{3-f}$	VT-OCP	Nonisothermal	−1.7(1)	−164(10)

[a]All data were collected using three-electrode setup with Pt working electrode, glass Ag/AgCl (filled with a 3 M aqueous KCl solution) or leak-free Ag/AgCl reference electrode, and Pt mesh counter electrode. [b]Isothermal and nonisothermal measurements were carried out using single-compartment and three-compartment electrochemical glass cells, respectively, as described in the Experimental Section. [c]Temperature coefficient (α) calculated using equations S1–S3, with the temperature coefficient of the reference electrode potential considered for measurements conducted under isothermal conditions; average values from 3–5 independent measurements and error bars denote standard deviations of those measurements. [d]Change in entropy associated with the given redox reaction, calculated using equation S4; errors were estimated using error propagation of the average temperature coefficient values. [e]Data collected using glass Ag/AgCl (filled with a 3 M aqueous KCl solution) reference electrode. [f]Data collected using leak-free Ag/AgCl reference electrode.

Table S4. Summary of select variable-temperature electrochemical studies of liquid-phase systems from the literature.[a]

Analyte	Method	Temperature Conditions	Solvent Type	Reference
$[Fe(CN)_6]^{3-}/[Fe(CN)_6]^{4-}$	VT-OCP	Isothermal[b]	Water	7
TM & Ln complexes[c]	VT-CV	Nonisothermal	Water	8
Mononuclear Fe(III) spin crossover complexes	VT-CV	Nonisothermal	Organic	9
TM complexes[d]	VT-CV	Nonisothermal	Water/Organic	10
TM complexes[e]	VT-CV	Nonisothermal	Water/Organic	11
TM complexes[f]	VT-CV	Nonisothermal	Water/Organic	12
Aromatic organic compounds	VT-PG[g]	Nonisothermal	Organic	13
Aromatic organic compounds	VT-PG[g]	Nonisothermal	Organic	14
Bis(biphenyl)chromium(I)	VT-PG[g]	Nonisothermal	Organic	15
Bis(biphenyl)chromium(I), ferrocene, perylene	VT-PG[g]	Nonisothermal	Organic	16
Br_2/Br^-	VT-OCP	Nonisothermal	Water	17
Mononuclear Fe complexes	VT-OCP	Nonisothermal	Ionic liquids	18
$[Fe(CN)_6]^{3-}/[Fe(CN)_6]^{4-}$	VT-OCP	Nonisothermal	Water	19
I_3^-/I^-	VT-OCP	Nonisothermal	Water/Organic/ Ionic liquids	20
Ferrocene-based compounds	VT-SWV[h]	Isothermal	Ionic liquids	21
Ferrocene-based compounds	VT-SWV[h]	Isothermal	Ionic liquids	22
I_3^-/I^-	VT-OCP	Nonisothermal	Ionic liquids	23
$[Co(bipy)_3]^{3+}/[Co(bipy)_3]^{2+}$[i]	VT-OCP	Nonisothermal	Ionic liquids	24
TM complexes[j]	VT-DPV[k]	Isothermal[l]	Ionic liquids	25
	VT-OCP	Nonisothermal		
Mono/di/polynuclear Fe(II) spin crossover complexes	VT-OCP	Nonisothermal	Organic	26
Tetrafluoro-*p*-quinone	VT-CV	Isothermal[l]	Water	27

$[Co(bipy)_3]^{3+}/[Co(bipy)_3]^{2+}$[i]	VT-OCP	Nonisothermal	Organic/ Ionic liquids	28
Mononuclear Co complexes	VT-OCP	Nonisothermal	Organic/ Ionic liquids	29
$[Fe(CN)_6]^{3-}/[Fe(CN)_6]^{4-}$	VT-OCP	Nonisothermal	Water	30
TM complexes[m]	VT-CV	Isothermal[l]	Water	31
	VT-OCP	Isothermal[l]		
Mononuclear TM spin crossover complexes[n]	VT-OCP	Nonisothermal	Organic	32
I_3^-/I^-	VT-OCP	Nonisothermal	Water	33
Polysulfide	VT-OCP	Nonisothermal	Organic	34
$[Fe(CN)_6]^{3-}/[Fe(CN)_6]^{4-}$	VT-OCP	Nonisothermal	Water	35
$[Fe(CN)_6]^{3-}/[Fe(CN)_6]^{4-}$	VT-OCP	Nonisothermal	Water	36
$[Ru(H_x im)_6]^{3+}/[Ru(H_x im)_6]^{2+}$[o]	VT-OCP	Nonisothermal	Water + Organic	37
	VT-SWV[h]	Isothermal[l]		
Mononuclear Fe^{2+} spin crossover complexes	VT-OCP	Nonisothermal	Organic	38
Mononuclear Fe complexes	VT-OCP	Nonisothermal	Water	39
$[Fe(CN)_6]^{3-}/[Fe(CN)_6]^{4-}$	VT-OCP	Nonisothermal	Water	40
$[Co(SARH_2)]^{5+}/[Co(SARH_2)]^{4+}$[p]	OCP[q]	Nonisothermal	Water/Organic/ Ionic liquids	41
Cobaltocene carboxylate and/or supramolecular hosts[r]	VT-OCP	Nonisothermal	Water	42
Wells–Dawson POM[s]	VT-CV	Isothermal[b]	Water/Organic	43
	VT-OCP	Nonisothermal		
$VO(OH)_3/[VO(H_2O)_3]^{2+}$	VT-OCP	Nonisothermal	Water	44
Keggin POMs[s]	VT-CV	Isothermal[l]	Organic	6
	VT-OCP	Nonisothermal		

[a]We direct interested readers to select review and perspective articles that highlight applications of redox electrolytes in electrochemical devices (refs 45–59). [b]Correction for the temperature dependence of the reference electrode potential was not mentioned. [c]Mononuclear transition metal and lanthanide complexes of Fe, Ru, Os, V, Cr, Co, Yb, and Eu were investigated. [d]Mononuclear transition metal complexes of Cr, Fe, and Co were investigated. [e]Mononuclear transition metal complexes of Ru and Co were investigated. [f]Mononuclear transition metal complexes of Fe, Ru, Os, Cr, and Co were investigated. [g]PG denotes polarography, which is a type of linear sweep voltammetry. [h]SWV denotes square-wave voltammetry. [i]bipy denotes 2,2′-bipyridine. [j]Mononuclear transition metal complexes of Fe, Ru, and Ni were investigated. [k]DPV denotes differential pulse voltammetry. [l]Values of temperature coefficients were corrected for the temperature dependence of the reference electrode potential. [m]Mononuclear transition metal complexes of Fe and Ag were investigated. [n]Octahedral complexes of Mn(II), Fe(II), and Co(II) with N-donor ligands were investigated. [o]Him denotes imidazole. [p]SAR denotes sarcophagine. [q]Temperature coefficients were determined from a single-point OCP measurement at a temperature gradient of 20 K. [r]Supramolecular hosts are tetrahedral M_4L_6 cages with M = Ga, Fe, In, Si, Ti, and Ge, and L denotes catecholate ligands. [s]POM denotes polyoxometalate.

E. References

(1) Lazar, M. A.; Al-Masri, D.; MacFarlane, D. R.; Pringle, J. M. Enhanced Thermal Energy Harvesting Performance of a Cobalt Redox Couple in Ionic Liquid–Solvent Mixtures. *Phys. Chem. Chem. Phys.* **2016**, *18* (3), 1404–1410.

(2) Bard, A. J.; Faulkner, L. R. *Electrochemical Methods: Fundamentals and Applications*, 2nd ed.; John Wiley & Sons, 2001.

(3) deBethune, A. J.; Licht, T. S.; Swendeman, N. The Temperature Coefficients of Electrode Potentials: The Isothermal and Thermal Coefficients—The Standard Ionic Entropy of Electrochemical Transport of the Hydrogen Ion. *J. Electrochem. Soc*. **1959**, *106* (7), 616–625.

(4) Greeley, R. S.; Smith, W. T., Jr.; Stoughton, R. W.; Lietzke, M. H. Electromotive Force Studies in Aqueous Solutions at Elevated Temperatures. I. The Standard Potential of the Silver–Silver Chloride Electrode. *J. Phys. Chem*. **1960**, *64* (5), 652–657.

(5) Kratochvil, B.; Lorah, E.; Garber, C. Silver–Silver Nitrate Couple as Reference Electrode in Acetonitrile. *Anal. Chem*. **1969**, *41* (13), 1793–1796.

(6) Dagar, M.; De, A.; Lu, Z.; Matson, E. M.; Thorarinsdottir, A. E. Implications of Charge and Heteroatom Dopants on the Thermodynamics and Kinetics of Redox Reactions in Keggin-Type Polyoxometalates. *ACS Mater. Au* **2025**, *5* (1), 200–210.

(7) Hanania, G. I. H.; Irvine, D. H.; Eaton, W. A.; George, P. Thermodynamic Aspects of the Potassium Hexacyanoferrate(III)–(II) System. II. Reduction Potential. *J. Phys. Chem*. **1967**, *71* (7), 2022–2030.

(8) Yee, E. L.; Cave, R. J.; Guyer, K. L.; Tyma, P. D., Weaver, M. J. A Survey of Ligand Effects upon the Reaction Entropies of Some Transition Metal Redox Couples. *J. Am. Chem. Soc*. **1979**, *101* (5), 1131–1137.

(9) Kadish, K. M.; Das, K.; Schaeper, D.; Merrill, C. L.; Welch, B. R.; Wilson, L. J. Spin State Dependent Redox Properties of the $[Fe^{III}(X\text{-}Sal)_2trien]^+$ Spin-Equilibrium System in Solution. *Inorg. Chem*. **1980**, *19* (9), 2816–2821.

(10) Sahami, S.; Weaver, M. J. Entropic and Enthalpic Contributions to the Solvent Dependence of the Thermodynamics of Transition-Metal Redox Couples: Part I. Couples Containing Aromatic Ligands. *J. Electroanal. Chem. Interfacial Electrochem*. **1981**, *122*, 155–170.

(11) Sahami, S.; Weaver, M. J. Entropic and Enthalpic Contributions to the Solvent Dependence of the Thermodynamics of Transition-Metal Redox Couples: Part II. Couples Containing Ammine and Ethylenediamine Ligands. *J. Electroanal. Chem. Interfacial Electrochem*. **1981**, *122*, 171–181.

(12) Hupp, J. T.; Weaver, M. J. Solvent, Ligand, and Ionic Charge Effects on Reaction Entropies for Simple Transition-Metal Redox Couples. *Inorg. Chem*. **1984**, *23* (22), 3639–3644.

(13) Jaworski, J. S. Entropy of Electrochemical Formation of *p*-Semiquinones in Dimethylformamide. *Monatsh. Chem*. **1986**, *117*, 151–157.

(14) Jaworski, J. S. Solvent and Ion Pairing Effects on the Reaction Entropy for the Electroreduction of Aromatic Molecules. *J. Electroanal. Chem*. *Interfacial Electrochem*. **1987**, *219* (1–2), 209–219.

(15) Jaworski, J. S. Reaction Entropy for the Electroreduction of Bis(biphenyl)chromium(I) in Different Solvents. *Polyhedron* **1987**, *6* (12), 2151–2153.

(16) Gritzner, G.; Lewandowski, A. Temperature Coefficients of Half-Wave Potentials and Entropies of Transfer of Cations in Aprotic Solvents. *J. Chem. Soc., Faraday Trans*. **1991**, *87* (16), 2599–2602.

(17) Shindo, K.; Arakawa, M.; Hirai, T. Influence of Electrode Materials on Open-Circuit Voltage Profiles with a Temperature Difference for a Thermocell Using a Br_2/Br^--Redox Reaction. *J. Power Sources* **2002**, *110* (1), 46–51.

(18) Migita, T.; Tachikawa, N.; Katayama, Y.; Miura, T. Thermoelectromotive Force of Some Redox Couples in an Amide-Type Room-Temperature Ionic Liquid. *Electrochemistry* **2009**, *77* (8), 639–641.

(19) Hu, R.; Cola, B. A.; Haram, N.; Barisci, J. N.; Lee, S.; Stoughton, S.; Wallace, G.; Too, C.; Thomas, M.; Gestos, A. et al. Harvesting Waste Thermal Energy Using a Carbon-Nanotube-Based Thermo-Electrochemical Cell. *Nano Lett*. **2010**, *10* (3), 838–846.

(20) Abraham, T. J.; MacFarlane, D. R.; Pringle, J. M. Seebeck Coefficients in Ionic Liquids –Prospects for Thermoelectrochemical Cells. *Chem. Commun*. **2011**, *47* (22), 6260–6262.

(21) Xiong, L.; Fletcher, A. M.; Ernst, S.; Davies, S. G.; Compton, R. G. An Electrochemical Thermometer: Voltammetric Measurement of Temperature and Its Application to Amperometric Gas Sensing. *Analyst* **2012**, *137* (11), 2567–2573.

(22) Xiong, L.; Fletcher, A. M.; Davies, S. G.; Norman, S. E.; Hardacre, C.; Compton, R. G. A Simultaneous Voltammetric Temperature and Humidity Sensor. *Analyst* **2012**, *137* (21), 4951–4957.

(23) Abraham, T. J.; MacFarlane, D. R.; Baughman, R. H.; Jin, L.; Li, N.; Pringle, J. M. Towards Ionic Liquid-Based Thermoelectrochemical Cells for the Harvesting of Thermal Energy. *Electrochim. Acta* **2013**, *113*, 87–93.

(24) Abraham, T. J.; MacFarlane, D. R.; Pringle, J. M. High Seebeck Coefficient Redox Ionic Liquid Electrolytes for Thermal Energy Harvesting. *Energy Environ. Sci*. **2013**, *6* (9), 2639–2645.

(25) Yamato, Y.; Katayama, Y.; Miura, T. Effects of the Interaction Between Ionic Liquids and Redox Couples on Their Reaction Entropies. *J. Electrochem. Soc*. **2013**, *160* (6), H309–H314.

(26) Abdullah, N.; Elsheikh, M. H.; Ibrahim, N. M. J. N.; Said, S. M.; Sabri, M. F. M.; Hassan, M. H.; Marlina, A. Magnetic, Thermal, Mesomorphic and Thermoelectric Properties of Mononuclear, Dimeric and Polymeric Iron(II) Complexes with Conjugated Ligands. *RSC Adv*. **2015**, *5* (63), 50999–51007.

(27) Yousofian-Varzaneh, H.; Zare, H. R.; Namazian, M. Thermodynamic Parameters and Electrochemical Behavior of Tetrafluoro-*p*-Quinone in Aqueous Solution. *J. Electrochem. Soc*. **2015**, *162* (8), G63–G68.

(28) He, J.; Al-Masri, D.; MacFarlane, D. R.; Pringle, J. M. Temperature Dependence of the Electrode Potential of a Cobalt-Based Redox Couple in Ionic Liquid Electrolytes for Thermal Energy Harvesting. *Faraday Discuss*. **2016**, *190*, 205–218.

(29) Al-Masri, D.; Dupont, M.; Yunis, R.; MacFarlane, D. R.; Pringle, J. M. The Electrochemistry and Performance of Cobalt-Based Redox Couples for Thermoelectrochemical Cells. *Electrochim. Acta* **2018**, *269*, 714–723.

(30) Duan, J.; Feng, G.; Yu, B.; Li, J.; Chen, M.; Yang, P.; Feng, J.; Liu, K.; Zhou, J. Aqueous Thermogalvanic Cells with a High Seebeck Coefficient for Low-Grade Heat Harvest. *Nat. Commun*. **2018**, *9*, No. 5146.

(31) Huang, B.; Muy, S.; Feng, S.; Katayama, Y.; Lu, Y.-C.; Chen, G.; Shao-Horn, Y. Non-Covalent Interactions in Electrochemical Reactions and Implications in Clean Energy Applications. *Phys. Chem. Chem. Phys*. **2018**, *20* (23), 15680–15686.

(32) Hasnan, M. M. I. M.; Abdullah, N.; Said, S. M.; Salleh, M. F. M.; Hussin, S. A. M.; Shah, N. M. Thermo-Electrochemical Generation Capabilities of Octahedral Spin Crossover Complexes of Mn(II), Fe(II) and Co(II) with N-Donor Ligands and Benzoate Counter Ion. *Electrochim. Acta* **2018**, *261*, 330–339.

(33) Liang, Y.; Yamada, T.; Zhou, H.; Kimizuka, N. Hexakis(2,3,6-tri-*O*-methyl)-α-cyclodextrin–I_5^- Complex in Aqueous I^-/I_3^- Thermocells and Enhancement in the Seebeck Coefficient. *Chem. Sci*. **2019**, *10* (3), 773–780.

(34) Liang, Y.; Hui, J. K.-H.; Yamada, T.; Kimizuka, N. Electrochemical Thermoelectric Conversion with Polysulfide as Redox Species. *ChemSusChem* **2019**, *12* (17), 4014–4020.

(35) Buckingham, M. A.; Hammoud, S.; Li, H.; Beale, C. J.; Sengel, J. T.; Aldous, L. A Fundamental Study of the Thermoelectrochemistry of Ferricyanide/Ferrocyanide: Cation, Concentration, Ratio, and Heterogeneous and Homogeneous Electrocatalysis Effects in Thermogalvanic Cells. *Sustain. Energy Fuels* **2020**, *4* (7), 3388–3399.

(36) Jung, S.-M.; Kwon, J.; Lee, J.; Shim, K.; Park, D.; Kim, T.; Kim, Y. H.; Hwang, S. J.; Kim, Y.-T. Cu-Based Thermoelectrochemical Cells for Direct Conversion of Low-Grade Waste Heat into Electricity. *ACS Appl. Energy Mater*. **2020**, *3* (7), 6383–6390.

(37) Kobayashi, T.; Yamada, T.; Tadokoro, M.; Kimizuka, N. A Novel Thermocell System Using Proton Solvation Entropy. *Chem. Eur. J*. **2021**, *27* (13), 4287–4290.

(38) Hassan, H. C.; Said, S. M.; Ibrahim, N. M. J. N.; Hasnan, M. M. I. M.; Noor, I. S. M.; Zakaria, R.; Salleh, M. F. M.; Noor, N. L. M.; Abdullah, N. Ultra-High Seebeck Coefficient of a Thermal Sensor Through Entropic Optimisation of Ligand Length of Fe(II) Spin-Crossover (SCO) Materials. *RSC Adv*. **2021**, *11* (34), 20970–20982.

(39) Buckingham, M. A.; Laws, K.; Cross, E.; Surman, A. J.; Aldous, L. Developing Iron-Based Anionic Redox Couples for Thermogalvanic Cells: Towards the Replacement of the Ferricyanide/Ferrocyanide Redox Couple. *Green Chem*. **2021**, *23* (22), 8901–8915.

(40) Jiang, L.; Kirihara, K.; Nandal, V.; Seki, K.; Mukaida, M.; Horike, S.; Wei, Q. Thermoelectrochemical Cells Based on Ferricyanide/Ferrocyanide/Guanidinium: Application and Challenges. *ACS Appl. Mater. Interfaces* **2022**, *14* (20), 22921–22928.

(41) Laws, K.; Buckingham, M. A.; Farleigh, M.; Ma, M.; Aldous, L. High Seebeck Coefficient Thermogalvanic Cells *via* the Solvent-Sensitive Charge Additivity of Cobalt 1,8-Diaminosarcophagine. *Chem. Commun*. **2023**, *59* (16), 2323–2326.

(42) Xia, K. T.; Rajan, A.; Surendranath, Y.; Bergman, R. G.; Raymond, K. N.; Toste, F. D. Tunable Electrochemical Entropy Through Solvent Ordering by a Supramolecular Host. *J. Am. Chem. Soc*. **2023**, *145* (46), 25463–25470.

(43) Svenson Grape, E.; Huang, J.; Roychowdhury, D.; Debela, T. T.; Chang, H.; Jenkins, A.; Schimpf, A. M.; Hendon, C. H.; Brozek, C. K. Converting Heat to Electrical Energy Using Highly Charged Polyoxometalate Electrolytes. *ACS Appl. Energy Mater*. **2024**, *7* (24), 11423–11428.

(44) Yamada, T.; Kobayashi, T.; Wakayama, Y.; Matoba, F.; Yatsuzuka, K.; Kimizuka, N.; Zhou, H. An Aqueous Vanadium Complex for the Superior Electrolyte of a Thermo-Electrochemical Cell. *Sustain. Energy Fuels* **2024**, *8* (4), 684–688.

(45) Quickenden, T. I.; Mua, Y. A Review of Power Generation in Aqueous Thermogalvanic Cells. *J. Electrochem. Soc*. **1995**, *142* (11), 3985–3994.

(46) Dupont, M. F.; MacFarlane, D. R.; Pringle, J. M. Thermo-Electrochemical Cells for Waste Heat Harvesting – Progress and Perspectives. *Chem. Commun*. **2017**, *53* (47), 6288–6302.

(47) Gao, C.; Lee, S. W.; Yang, Y. Thermally Regenerative Electrochemical Cycle for Low-Grade Heat Harvesting. *ACS Energy Lett*. **2017**, *2* (10), 2326–2334.

(48) Buckingham, M. A.; Aldous, L. Thermogalvanic Cells: A Side-by-Side Comparison of Measurement Methods. *J. Electroanal. Chem*. **2020**, *872*, No. 114280.

(49) Cheng, C.; Dai, Y.; Yu, J.; Liu, C.; Wang, S.; Feng, S. P.; Ni, M. Review of Liquid-Based Systems to Recover Low-Grade Waste Heat for Electrical Energy Generation. *Energy Fuels* **2021**, *35* (1), 161–175.

(50) Liu, W.; Qian, X.; Han, C.-G.; Li, Q.; Chen, G. Ionic Thermoelectric Materials for Near Ambient Temperature Energy Harvesting. *Appl. Phys. Lett*. **2021**, *118* (2), No. 020501.

(51) Hasan, M. A. M.; Wu, H.; Yang, Y. Redox-Induced Electricity for Energy Scavenging and Self-Powered Sensors. *J. Mater. Chem. A* **2021**, *9* (35), 19116–19148.

(52) Duan, J.; Yu, B.; Huang, L.; Hu, B.; Xu, M.; Feng, G.; Zhou, J. Liquid-State Thermocells: Opportunities and Challenges for Low-Grade Heat Harvesting. *Joule* **2021**, *5* (4), 768–779.

(53) Liu, Y.; Wang, H.; Sherrell, P. C.; Liu, L.; Wang, Y.; Chen, J. Potentially Wearable Thermo-Electrochemical Cells for Body Heat Harvesting: From Mechanism, Materials, Strategies to Applications. *Adv. Sci*. **2021**, *8* (13), No. 2100669.

(54) Zhang, H.; Wang, Q. Thermally Regenerative Electrochemical Cycle for Low-Grade Heat Harnessing. *Chem. Phys. Rev*. **2021**, *2* (2), No. 021304.

(55) Li, M.; Hong, M.; Dargusch, M.; Zou, J.; Chen, Z.-G. High-Efficiency Thermocells Driven by Thermo-Electrochemical Processes. *Trends Chem*. **2021**, *3* (7), 561–574.

(56) Liu, Y.; Cui, M.; Ling, W.; Cheng, L.; Lei, H.; Li, W.; Huang, Y. Thermo-Electrochemical Cells for Heat to Electricity Conversion: From Mechanisms, Materials, Strategies to Applications. *Energy Environ. Sci*. **2022**, *15* (9), 3670–3687.

(57) Zhou, H.; Inoue, H.; Ujita, M.; Yamada, T. Advancement of Electrochemical Thermoelectric Conversion with Molecular Technology. *Angew. Chem., Int. Ed*. **2023**, *62* (2), No. e202213449.

(58) Qian, X.; Ma, Z.; Huang, Q.; Jiang, H.; Yang, R. Thermodynamics of Ionic Thermoelectrics for Low-Grade Heat Harvesting. *ACS Energy Lett*. **2024**, *9* (2), 679–706.

(59) Li, Z.; Jiang, J.; He, X.; Wang, C.; Niu, Y. Recent Progress on the Thermoelectric Effect for Electrochemistry. *J. Mater. Chem. A* **2024**, *12* (23), 13623–13646.
